# NR2E3 loss disrupts photoreceptor cell maturation and fate in human organoid models of retinal development

**DOI:** 10.1172/JCI173892

**Published:** 2024-04-23

**Authors:** Nathaniel K. Mullin, Laura R. Bohrer, Andrew P. Voigt, Lola P. Lozano, Allison T. Wright, Vera L. Bonilha, Robert F. Mullins, Edwin M. Stone, Budd A. Tucker

**Affiliations:** 1 Institute for Vision Research and; 2 Department of Ophthalmology and Visual Sciences, Carver College of Medicine, University of Iowa, Iowa City, Iowa, USA.; 3 Department of Ophthalmic Research, Cleveland Clinic, Cleveland, Ohio, USA.; 4 Department of Ophthalmology, Cleveland Clinic Lerner College of Medicine, School of Medicine, Case Western Reserve University, Cleveland, Ohio, USA.

**Keywords:** Development, Ophthalmology, Genetic diseases, Monogenic diseases, iPS cells

## Abstract

While dysfunction and death of light-detecting photoreceptor cells underlie most inherited retinal dystrophies, knowledge of the species-specific details of human rod and cone photoreceptor cell development remains limited. Here, we generated retinal organoids carrying retinal disease–causing variants in *NR2E3*, as well as isogenic and unrelated controls. Organoids were sampled using single-cell RNA sequencing (scRNA-Seq) across the developmental window encompassing photoreceptor specification, emergence, and maturation. Using scRNA-Seq data, we reconstruct the rod photoreceptor developmental lineage and identify a branch point unique to the disease state. We show that the rod-specific transcription factor NR2E3 is required for the proper expression of genes involved in phototransduction, including rhodopsin, which is absent in divergent rods. NR2E3-null rods additionally misexpress several cone-specific phototransduction genes. Using joint multimodal single-cell sequencing, we further identify putative regulatory sites where rod-specific factors act to steer photoreceptor cell development. Finally, we show that rod-committed photoreceptor cells form and persist throughout life in a patient with NR2E3-associated disease. Importantly, these findings are strikingly different from those observed in Nr2e3 rodent models. Together, these data provide a road map of human photoreceptor development and leverage patient induced pluripotent stem cells to define the specific roles of rod transcription factors in photoreceptor cell emergence and maturation in health and disease.

## Abstract 2

Single-cell sequencing of patient-derived retinal organoids carrying genetic variants within NR2E3 reveals the roles of this transcription factor in normal development and disease.

## Graphical abstract

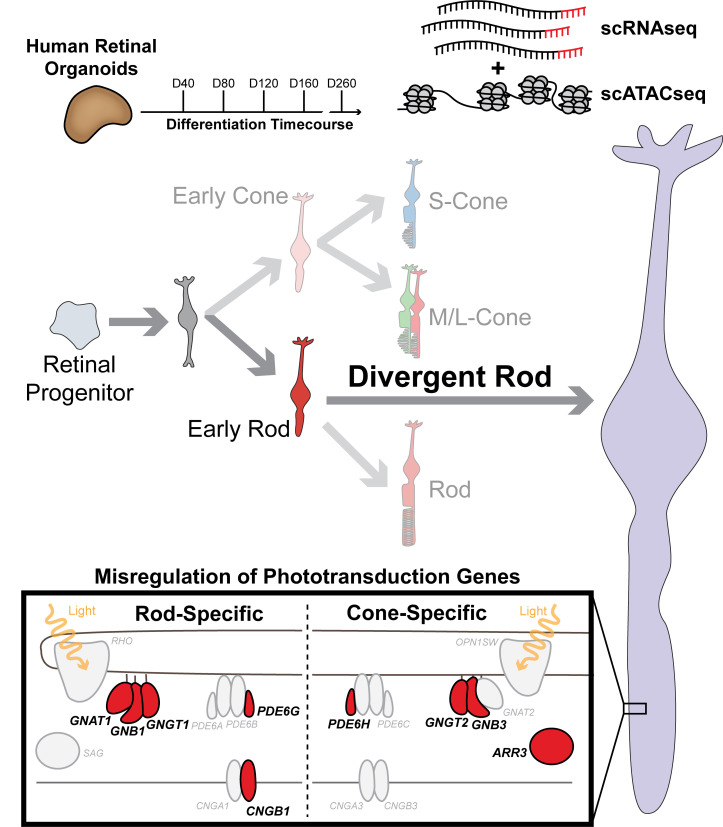

## Introduction

The human retina is a transparent multilayered neural tissue lining the posterior two-thirds of the eye that is responsible for detecting visual information and sending it to the brain via the second cranial nerve. Light-detecting photoreceptor cells, which make up the outermost layer of the neural retina, emerge during early development from a pool of multipotent neural progenitors (1, 2). In humans, death of photoreceptor cells, which is associated with inherited retinal degenerative disease, is a major cause of incurable blindness. Disease-causing variants in genes such as *USH2A*, *RHO*, and *RPGR*, which are the leading causes of recessive, dominant, and X-linked disease, respectively, result in progressive vision loss typically beginning in late teens and young adults (3). The relatively late onset and slowly progressive nature of these disorders allow for real-time investigation using a variety of different clinical approaches. For transcription factor genes that regulate photoreceptor cell fate commitment and maturation, clinical evaluation of the early disease state is often impossible. That is, mutations in transcription factor genes often result in the absence of specific cell types at birth (4–8). Understanding the precise role of transcription factors in human photoreceptor cell development and how loss-of-function mutations cause disease has the potential to novel approaches to rescue dysfunctional photoreceptors in patients diagnosed with an inherited retinal degeneration.

Mutations in the nuclear receptor subfamily 2 group E member 3 (*NR2E3*) gene cause enhanced S-cone syndrome (ESCS) (OMIM 268100), a congenital retinal disease characterized by night blindness, hypersensitivity to short-wavelength light, and eventual loss of visual acuity (9, 10). Rod photoreceptors are the primary effectors of vision in dim light. *NR2E3* is expressed in rod photoreceptors and plays an essential role in rod development in concert with upstream rod transcription factors including neural retina leucine zipper (NRL) (11). The retinas of ESCS patients additionally exhibit disorganization of the normal cellular layers (12). Postmortem histological observation of ESCS patients’ retinas has demonstrated a lack of staining for rhodopsin, the primary functional molecule of light sensitivity in rod photoreceptors (12). Additionally, there are an increased proportion of cones, specifically S-cones, which mislocalize into the layers of the retina typically restricted to rod photoreceptors (12). Much of the knowledge of mammalian rod photoreceptor specification comes from murine models (13). The Rd7 mouse harbors spontaneous mutations in *Nr2e3*, which cause a retinal degeneration phenotype reminiscent of ESCS patients (14). However, key rod function genes such as that encoding the light-sensitive protein rhodopsin (*Rho*) are expressed in the Nr2e3-deficient mouse retina (14–17), in contrast to the complete loss of rod function observed in ESCS patients. As the mouse has a rod-dominant retina lacking a cone-rich macula (18), and the requirement of core rod transcription factors for rod specification is known to vary in other vertebrates (19), the precise regulatory processes governing rod and cone photoreceptor specification and maturation may differ between species. Thus it is essential to study human retinal cells to understand the pathogenesis of *NR2E3*-associated disease and related conditions.

To define the specific timing and targets of NR2E3 activity in human retinal development, we performed transcriptome, chromatin, and protein-level analysis across a 260-day time course from early retinal commitment through photoreceptor cell maturation. We used an effectively NR2E3-null human induced pluripotent stem cell (iPSC) line derived from a clinically diagnosed ESCS patient. To control for the effects of genetic background on organoid differentiation efficiency, we differentiated 2 control lines in parallel: a CRISPR-corrected isogenic control and an unrelated healthy donor control. We used single-cell RNA sequencing (scRNA-Seq) to identify a population of rod photoreceptor cells (which we categorize as “divergent”) that emerged between differentiation days 80 and 120 in the NR2E3-null organoids. We showed that these cells, which persist throughout retinal development, coexpress rod and cone photoreceptor cell markers that are typically expressed in mature cells at both the transcript and protein levels. We further show that these cells formed and persisted in the retina of a patient with severe NR2E3-associated retinal dystrophy. Interestingly, after 120 days of differentiation, the majority of divergent rod photoreceptors were refractory to rescue by wild-type NR2E3 supplementation, which highlights the temporal requirement of this transcription factor for rod photoreceptor cell development. Using single-cell multimodal sequencing, we showed that misregulation of these genes is due to changes in the activity of *cis*-regulatory elements following loss of NR2E3 function. Interestingly, at later stages of retinal development an increase in the number of blue cones at the expense of divergent rods was detected. That is, while divergent rods persisted throughout development, their proportion decreased as the proportion of blue cones increased, which may suggest a divergent rod to blue cone conversion. Together, these data define a specific role for *NR2E3* in human photoreceptor cell development that appears to be distinct from that of rodents, which sheds light on the cellular changes underlying the ESCS retinal phenotype.

## Results

### Retinal organoids produce developmentally timed cell types.

**Figure 1:**
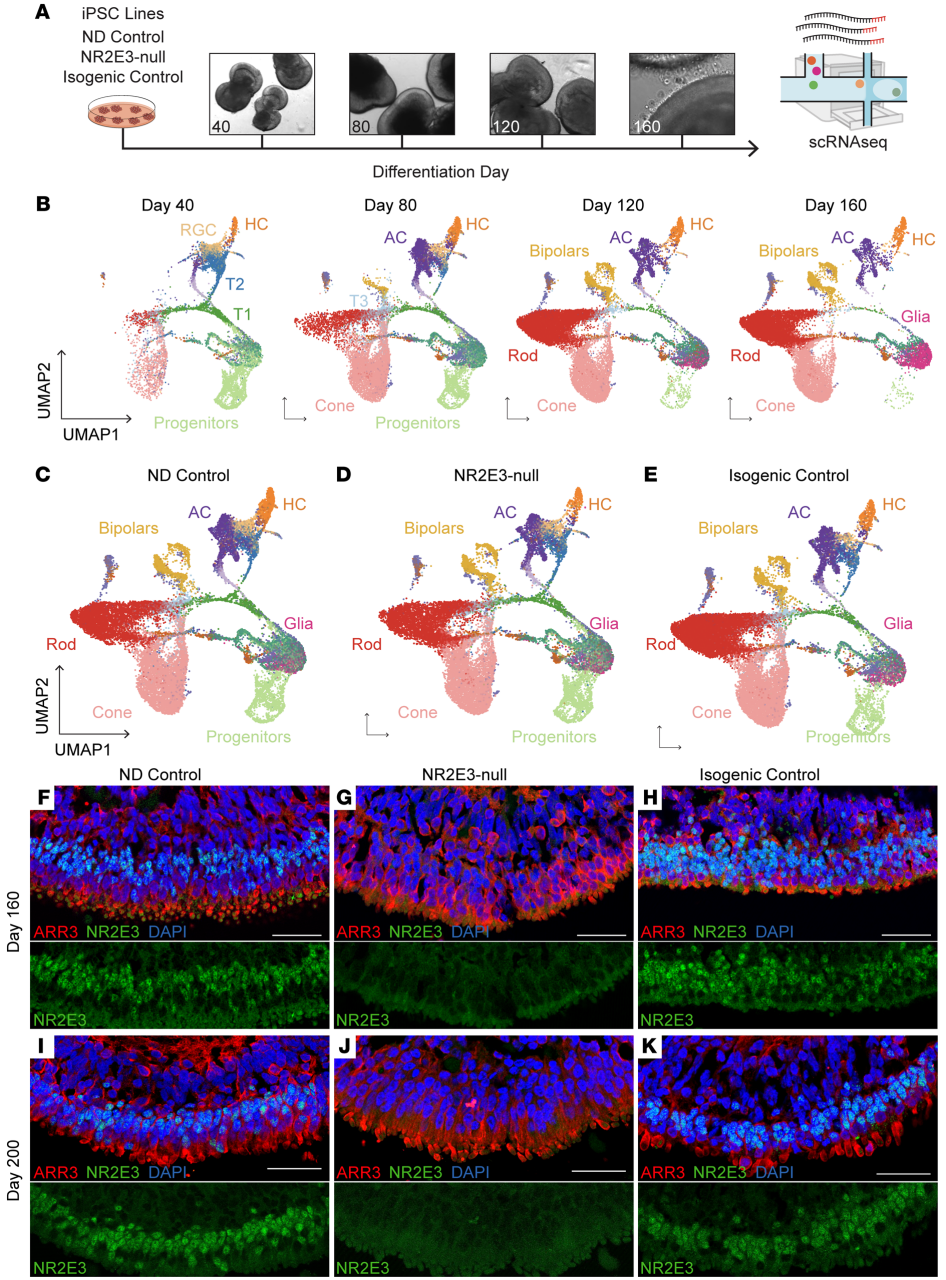
Modeling pathological retinal development using retinal organoids. (**A**) Schematic for organoid differentiation time course with scRNA-Seq. (**B**) Integrated and annotated cells recovered from all scRNA-Seq samples projected in 2D space using UMAP embeddings. Cells are grouped by time point of collection. (**C**–**E**) Cells from all time points are split by cell line of origin. (**F**) No-disease control (ND control) organoids express NR2E3 (green) in rod nuclei at D160 of differentiation. Cone photoreceptors express cone arrestin (ARR3, red).(**G**) NR2E3-null organoids express cone arrestin but lack expression of NR2E3. (**H**) Monoallelic correction of NR2E3 restores expression of NR2E3 in D160 organoids. (**I**–**K**) At D200 of differentiation, NR2E3 expression remains high in ND control and isogenic control lines and is absent in NR2E3-null samples. Scale bars: 50 μm.

To determine how and when NR2E3 acts in human retinal development, we used a previously described iPSC line (20) derived from an ESCS patient with a homozygous c.119-2A>C mutation in *NR2E3*. This mutation causes the inclusion of a portion of intron 1, which creates a frameshift and premature stop codon following the first exon, rendering it null (referred to as NR2E3-null going forward). We previously showed that monoallelic correction of c.119-2A>C in patient-derived iPSCs by CRISPR-mediated homology-dependent repair restores the ability of photoreceptor cells to make wild-type *NR2E3* transcript during retinal cell differentiation (20). To capture both developing and terminally differentiated cell types, retinal organoids were generated from no-disease control, NR2E3-null, and CRISPR-corrected NR2E3 (isogenic control) lines, and these organoids were initially sampled across a 160-day time course (Figure 1A). iPSC lines were characterized for pluripotency and genomic stability (Supplemental Figure 1; supplemental material available online with this article; https://doi.org/10.1172/JCI173892DS1), and the full *NR2E3* locus of the no-disease control was sequenced to confirm the absence of potentially deleterious variants (Supplemental Figure 1J). Organoids were assayed using single-cell transcriptome profiling and immunohistochemistry (Figure 1 and Supplemental Figure 2). Data from cells collected on days 40, 80, 120, and 160 (hereafter referred to as D40, D80, D120, and D160) of differentiation across all 3 lines were aggregated and annotated using previously published human organoid and fetal retina scRNA-Seq data (21). Cell type emergence followed the known developmental cadence of retinal formation (1), with progenitors giving rise to cone photoreceptors and inner retinal cells first, followed by waves of rod photoreceptor and bipolar cell emergence (Figure 1B). Notably, all expected cell types, including rod photoreceptors, were observed in each line (Figure 1, C–E).

NR2E3 is required for normal rod photoreceptor development (12, 22, 23) and is known to be expressed soon after the induction of its upstream activator, NRL (24). We observed the emergence of rod photoreceptors in organoids by D120 of differentiation in all 3 lines (Figure 1, B–E). We next stained for the NR2E3 protein in fixed sections of retinal organoids at comparable time points. Control organoids expressed NR2E3 in the nucleus at D160 (Figure 1F). No NR2E3 protein was detected in the NR2E3-null organoids at the same time point (Figure 1G), and monoallelic correction of the locus restored normal expression (Figure 1H). The same pattern of protein expression persisted to D200 (Figure 1, I–K), indicating that the c.119-2A>C *NR2E3* mutations cause a total lack, rather than delay, of NR2E3 protein expression in human retinal organoids.

### Divergent rods emerge in NR2E3-null organoids following rod commitment.

**Figure 2:**
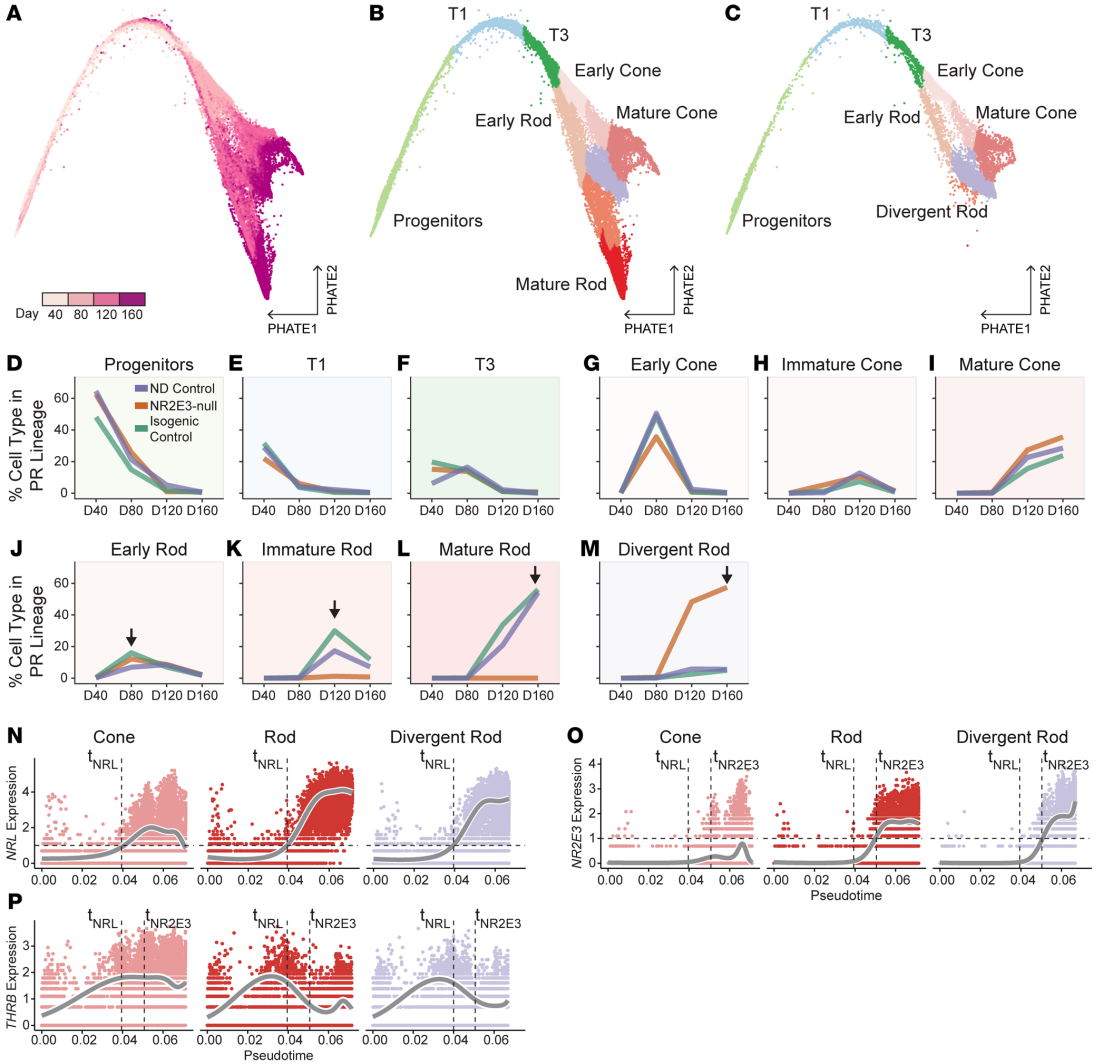
Divergent rods emerge in NR2E3-null organoids. (**A**) PHATE reduction showing cells within the photoreceptor lineage. Cells are colored by time point of sample collection. (**B**) Cells from NR2E3-null and control lines are annotated together based on time point and PHATE-derived cluster. (**C**) Cells annotated based on PHATE clustering from only the NR2E3-null line. (**D**–**F**) The proportion of early and intermediate progenitors decreases uniformly across differentiation of all lines. (**G**–**I**) The proportion of maturing cones follows differentiation time point in all lines. (**J**) All lines form early rod photoreceptors at D80 (arrow). (**K** and **L**) Only ND control and isogenic control lines form immature and mature rod photoreceptors at D120 and D160 (arrows). (**M**) Divergent rods emerge by D120 and are largely restricted to the NR2E3-null line (arrow). (**N**) *NRL* expression is plotted against pseudotime for each lineage on a log scale. *NRL* expression is observed at comparable levels in rod and divergent rod lineages and is induced at the same point in pseudotime. The pseudotime value at which *NRL* expression passes 1 is shown as t_NRL_. (**O**) *NR2E3* expression level across pseudotime is shown. In addition to t_NRL_ (*NRL* induction pseudotime point), the point at which *NR2E3* expression passes 1 is shown as t_NR2E3_. The timing of NR2E3 induction is similar in rod and divergent rod lineages. (**P**) *THRB* expression level across pseudotime is shown.

Since NR2E3 is known to be required for rod photoreceptor cell formation, we next asked how NR2E3-null rods differed transcriptionally from normal rod photoreceptors. We computationally isolated the photoreceptor lineage within the data set to enable comparison of developmental lineages of rod and cone photoreceptors. D40, D80, D120, and D160 cells from all 3 lines that were annotated as Progenitors, T1, T3, Cone, or Rod (i.e., from Figure 1B) were reprocessed using potential of heat diffusion for affinity-based transition embedding (PHATE) (25), a dimensionality-reduction technique suited to maintaining the branching structure in developmental data sets. The ordering of cells in the PHATE embedding matched collection time points of the samples (Figure 2A), lending confidence to the biological relevance of this approach. Cells were reclustered and manually reannotated based on the PHATE embedding (Figure 2B) using time point and expression information of marker genes (Supplemental Figure 3A). In addition to refining the maturity of rod and cone photoreceptors (e.g., early cone, immature cone, cone), a novel cluster was also observed in the PHATE reduction that appeared largely restricted to the NR2E3-null cells (Figure 2C). Since it branched from the early rod cluster, we named these cells “divergent rods.”

To better understand the developmental origin of the divergent rod cluster, we calculated the proportion of each cell type at each time point of photoreceptor lineage differentiation (Figure 2, D–M). When cell type proportions are plotted across time, the disappearance of progenitors followed by the emergence of maturing photoreceptor cells is seen (Figure 2, D–L), indicating that proper commitment and maturation of cell types occur within retinal organoids of all 3 lines. However, emergence of normally mature rods is observed only in control lines (Figure 2, K and L), while formation of divergent rods is restricted to the NR2E3-null line (orange line) at D120 (Figure 2M). Notably, the NR2E3-null line produces early rods at D80 at roughly similar proportions to control lines (Figure 2J), counter to the hypothesis that NR2E3-null photoreceptor progenitors would be uniformly shunted into a cone cell fate prior to this developmental time point. Instead, these data support rod malformation in NR2E3-null as occurring after rod photoreceptor cell fate commitment at D80.

To further investigate the differences between normal and pathological rod differentiation, we next identified 3 trajectories through the PHATE embedding using Slingshot (26) (Supplemental Figure 3, B–E). These trajectories describe the maturation of progenitor cells into normal cones, normal rods, or divergent rods. Trajectories were used to compute pseudotime values for each cell within each lineage (Supplemental Figure 3, C–H). We plotted expression of *NRL* against pseudotime within each lineage (Figure 2N) and observed comparable timing and level of *NRL* expression in the normal and divergent rod lineages. NRL levels were markedly lower in the cone lineage. We generated similar plots for *NR2E3* (Figure 2O) that show induction of *NR2E3* expression following *NRL* induction in only the rod and divergent rod trajectories. The cone-specifying factor *THRB* followed a similar expression pattern in rod and divergent rod lineages, indicating that they did not acquire cone fate at the time point of normal *NR2E3* induction (Figure 2P). These findings suggest that NRL-expressing divergent rods initially develop normally and that a failure in rod maturation later in development can be attributed to NR2E3 loss.

### Joint multimodal sequencing of divergent rod transcriptome and chromatin accessibility.

**Figure 3:**
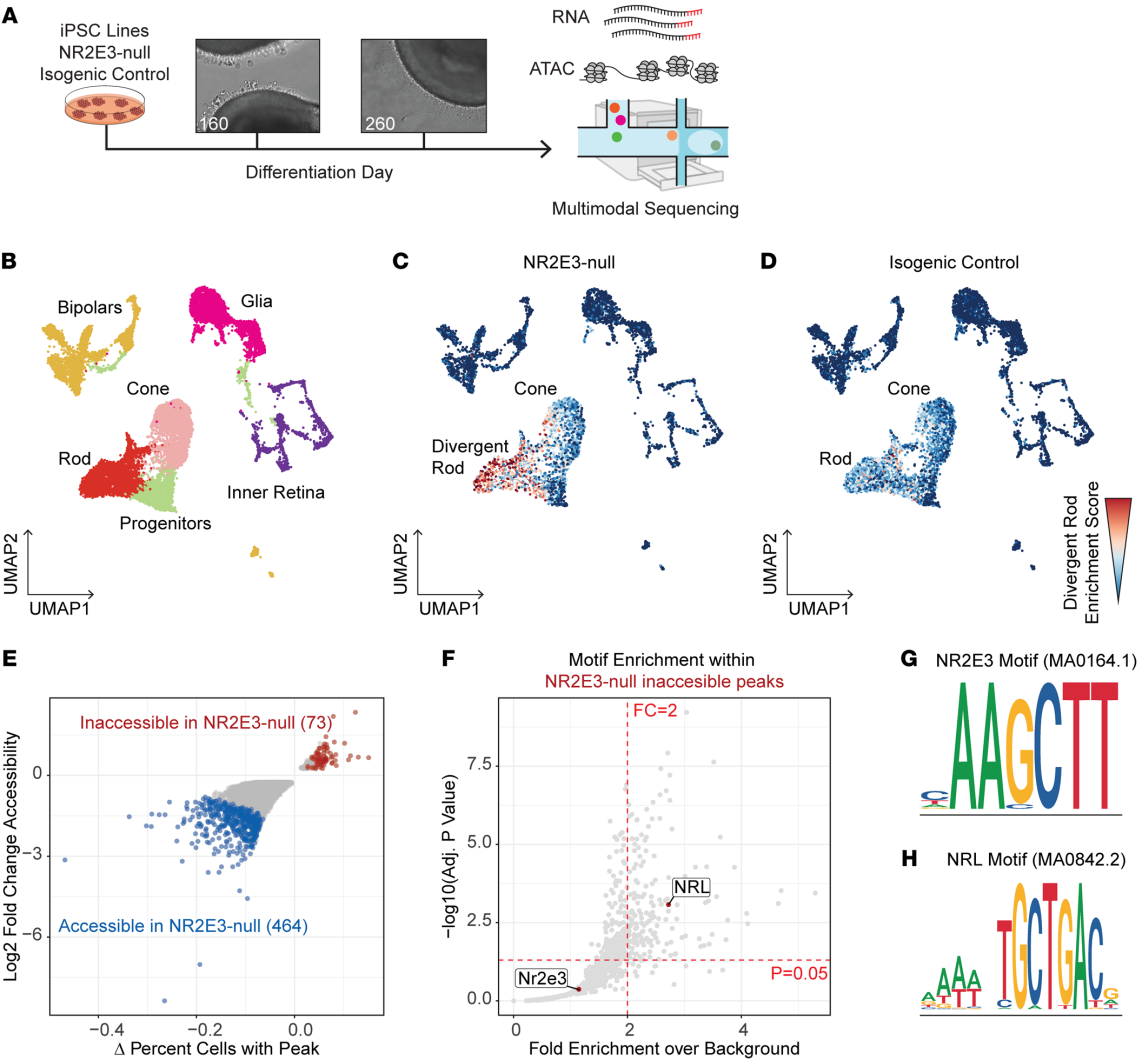
NR2E3 loss disrupts rod chromatin accessibility. (**A**) Experimental schematic showing collection of nuclei from D160 and D260 retinal organoids for joint multimodal single-nucleus sequencing. (**B**) Annotated WNN-UMAP projections of cells assayed by joint multimodal single-nucleus sequencing. Both lines contribute to all cell type clusters. (**C** and **D**) Two-dimensional projection of cells based on WNN analysis of gene expression and ATAC-seq profiles. Cells split by line (NR2E3-null and isogenic control). Cells are shaded based on divergent rod gene module score, with red indicating enrichment for divergent rod module genes. (**E**) Differential ATAC peak accessibility between NR2E3-null and isogenic control rods. Peaks that are more accessible in the control line (i.e., closed in the NR2E3-null rods) are shown in red, while peaks that are more accessible in the NR2E3-null line are shown in blue. More peaks are accessible in NR2E3-null versus control, indicating a globally repressive role for NR2E3 in maturing rod photoreceptors. (**F**) Transcription factor binding motif enrichment in peaks that are inaccessible in the NR2E3-null rods versus control. Enrichment of the NRL motif indicates reliance of NRL on NR2E3 presence for binding. (**G** and **H**) Motif symbols for the NR2E3- and NRL-binding motifs used for analysis in **F**.

To confirm that the emergence of divergent rods was not an artifact of batch-to-batch variability of organoid differentiation and to gain information on chromatin remodeling following NR2E3 loss in rods, we performed single-nucleus multimodal sequencing on retinal organoid nuclei isolated from an independent round of differentiation (Figure 3A) of the NR2E3-null and isogenic control lines. Nuclei were collected from time points after the emergence of divergent rods (D160 and D260). Joint multimodal RNA and assay for transposase-accessible chromatin (ATAC) sequencing were performed to query the differential gene expression and accessibility of regulatory regions in NR2E3-null rods. First, nuclei were clustered and annotated on a weighted combination of ATAC and gene expression modalities (Figure 3B). Cone and rod photoreceptor nuclei were captured in both NR2E3-null and isogenic control organoids at D160 and D260, recapitulating the finding of our previous time course study (Figures 1 and 2). Using the D40–D160 gene expression data from the photoreceptor lineage (Figure 2, B and C), rod, cone, and divergent rod gene modules were computed (Supplemental Figure 4, A–J). Nuclei in the multimodal data set were scored for each module. An enrichment within the NR2E3-null rod cluster for the divergent rod gene module was observed (Figure 3, C and D, and Supplemental Figure 4, G–I). This enrichment was not observed in isogenic control rods (Figure 3C and Supplemental Figure 4, H and I). These data show that the emergence of divergent rods in NR2E3-null organoids is reproducible and robust to discovery across different sequencing modalities and rounds of differentiation.

### NR2E3 acts as a direct suppressor of cone-specific gene expression.

Since Nr2e3 acts as a suppressor of cone photoreceptor cell fate in mice (23), we were interested in how loss of NR2E3 function in human iPSC-derived photoreceptors altered the chromatin accessibility around cone genes and caused their misregulation. After ATAC peaks were called, differentially accessible regions (DARs) between the NR2E3-null and isogenic control lines within the rod cluster were identified (Figure 3E). Notably, far more peaks were accessible in the NR2E3-null sample, indicating NR2E3’s globally repressive role (464 peaks preferentially accessible in NR2E3-null compared with control, with 73 peaks preferentially inaccessible in the NR2E3-null cells). Several of these DARs were near rod- and cone-specific genes, indicating a dual role for NR2E3 in cone gene repression and rod gene activation (Supplemental Figure 4K). We computed enrichment of known transcription factor binding motifs within ATAC peaks that were inaccessible in NR2E3-null rod photoreceptors (Figure 3F). Of specific interest were the canonical NRL- and NR2E3-binding motifs (Figure 3, G and H). The NRL-binding motif (TGCTGAC) was statistically significantly enriched in the set of peaks that become inaccessible following NR2E3 loss, while the NR2E3-binding motif was not (Figure 3F). This shows that NRL binding and subsequent chromatin remodeling require the presence of NR2E3 in at least some contexts.

### Divergent rods misexpress cone- and rod-specific phototransduction genes.

**Figure 4:**
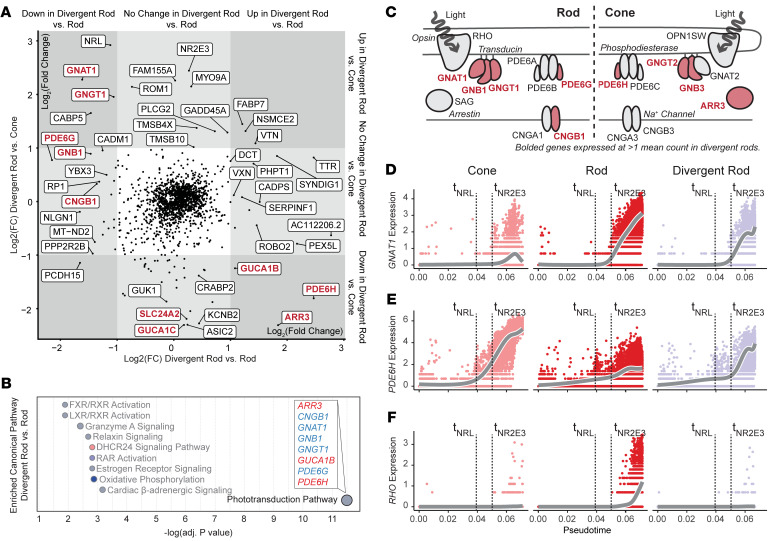
Divergent rods express a combination of rod- and cone-specific genes involved in phototransduction. (**A**) Differentially expressed genes between the divergent rod and rod (*x* axis) or cone (*y* axis) lineages. Compared with normal rods, divergent rods upregulate several cone-specific transcripts, as well as genes involved in synaptogenesis. Compared with normal cones, divergent rods upregulate canonical rod transcripts. Genes involved in phototransduction are highlighted in red. (**B**) Diagram of rod-specific (left) and cone-specific (right) components of the phototransduction pathway. Genes expressed in divergent rods are colored, and those not expressed in divergent rods are shown in gray. (**C**) Pathway enrichment analysis for differentially expressed genes between divergent rod and rod clusters (the *x* axis of **A**). (**D**) The rod-specific transducin component (*GNAT1*) is expressed in rod and divergent rod lineages but not in normal cones. (**E**) The cone-specific phosphodiesterase *PDE6H* is expressed in the normal cone lineage and in divergent rods across the same developmental time. (**F**) The rod-specific opsin *RHO* is expressed late in normal rod development but not divergent rods. For **D**–**F**, t_NRL_ and t_NR2E3_ indicate pseudotime points of NRL and NR2E3 expression induction, respectively (as in Figure 2, O and P).

We next asked how the gene expression pattern of divergent rods differed from that of normal rods and cones. A differential expression test was performed in a pairwise fashion between the most mature clusters of each of 3 lineages (rods, cones, and divergent rods; see Figure 2, B and C). Remarkably, several well-described functional photoreceptor genes were misexpressed in divergent rods (Figure 4A, highlighted in red). Comparison of gene expression between NR2E3-null and isogenic control cells showed that dysregulation was restricted to rods (Supplemental Figure 5, A–N). Normal expression patterns of rod- and cone-specific genes were confirmed using scRNA-Seq data from human donor retina (27) (Supplemental Figure 5, O–V). These data indicate that these divergent rods retain rod identity and are not shunted to a cone fate in early differentiation; instead, they exist as fate-committed photoreceptor cells expressing a combination of rod and cone genes.

Differentially expressed genes between divergent rods and normal rods were subjected to pathway enrichment analysis to better understand the cellular changes downstream of NR2E3 loss. The top enriched pathway in the divergent rod differentially expressed genes was “phototransduction pathway” (Figure 4B), validating the observed misexpression pattern of genes such as *ARR3*, *PDE6H*, *GNAT1*, and *GNGT1* in Figure 4A. Divergent rods expressed a combination of rod- and cone-specific phototransduction genes but failed to express either rod or cone opsin (Figure 4C). The expression of phototransduction genes along pseudotime in divergent rods compared with the normal rod and cone lineages showed that misexpression followed normal timing (Figure 4, D–F). Specifically, expression of cone genes in divergent rods temporally occurred in accordance with expression in normal cones, and expression of rod genes in divergent rods did so in accordance with normal rods. Upregulation of *PDE6H* in divergent rods occurred only after the time point at which NR2E3 would normally act to suppress this cone-specific gene (Figure 4E). Based on these analyses, NR2E3 loss in developing photoreceptors causes misexpression of cone- and rod-specific genes involved in phototransduction, the major function of photoreceptor cells in reception and processing of visual information.

**Figure 5:**
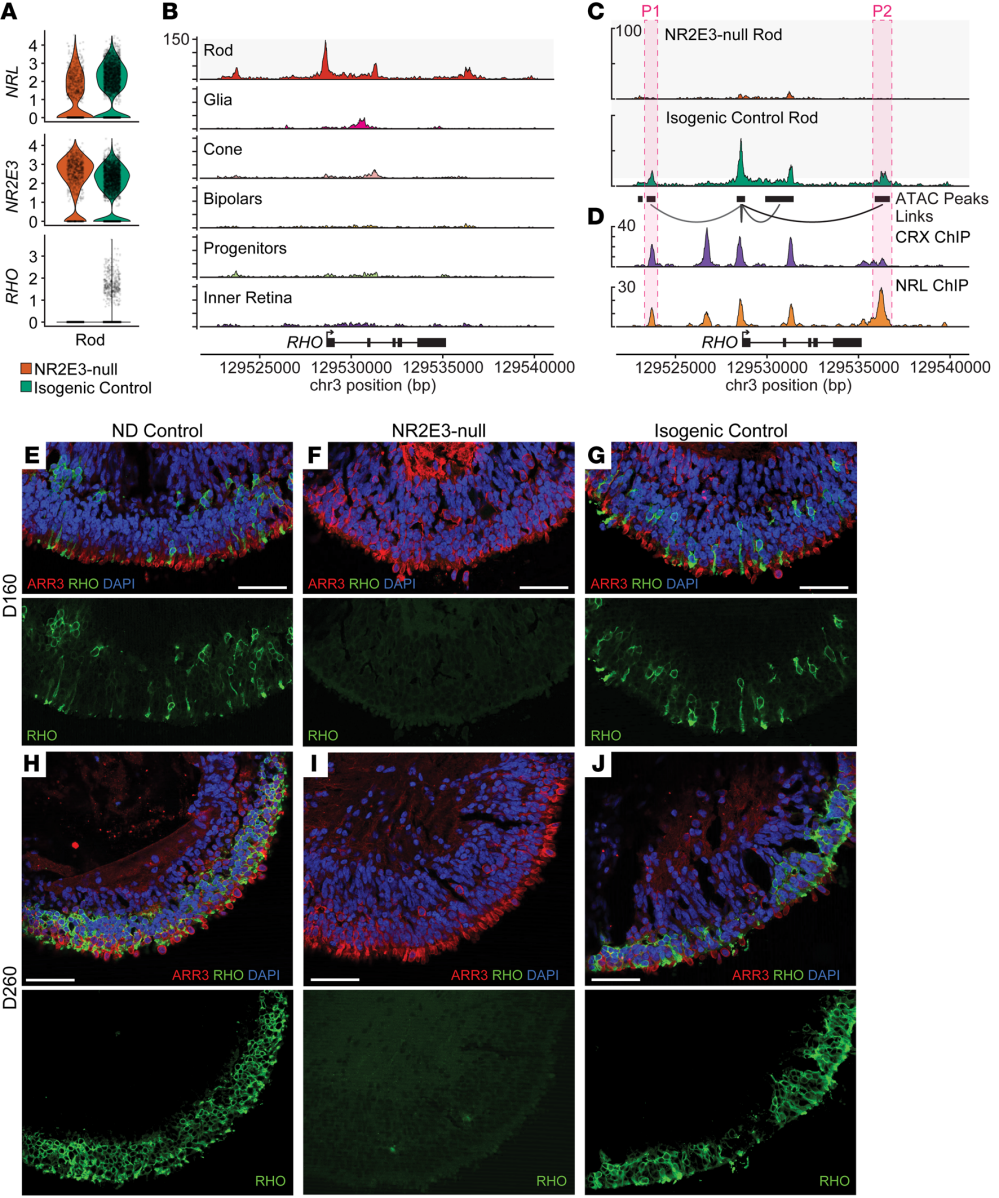
NR2E3-null rods fail to activate expression of rhodopsin. (**A**) Violin plots show expression of *NRL*, *NR2E3*, and *RHO* within rods from either NR2E3-null or isogenic control organoids (D120 and D160 combined from the multimodal sequencing experiment in Figure 2). NR2E3-null rods express the transcription factors *NRL* and *NR2E3* at the transcript level but do not express *RHO* transcript. (**B**) ATAC coverage tracks for isogenic control organoids (D160 and D260 combined) are shown. Accessibility in regions around *RHO* is observed in the rod cluster. (**C**) ATAC coverage for the rod cluster is shown for NR2E3-null and isogenic control samples. Below coverage tracks, ATAC peaks are shown as black boxes. Lines connecting peaks to the transcriptional start site of *RHO* represent peak-to-gene linkages. Two peaks (P1 and P2) that are linked to *RHO* expression and accessible only in control rods are highlighted in red. (**D**) CRX and NRL ChIP-Seq tracks from adult human donor eye samples are shown aligned to the tracks in **C**. NR2E3-dependent peaks highlighted in **C** are bound by NRL in human retina. (**E**–**G**) At D160, *RHO*-expressing photoreceptors are observed in ND control (**E**) and isogenic control (**G**) organoids, but no *RHO*-expressing cells are seen in NR2E3-null organoids (**F**). (**H**–**J**) By D260, *RHO* expression increases in ND control (**H**) and isogenic control (**J**) organoids but is still absent from NR2E3-null organoids (**I**). Scale bars: 50 μm.

To investigate the potential functionality of divergent rods, we next examined expression of the rod-specific opsin gene *RHO*. While divergent rods express *NRL* and the variant-containing *NR2E3* at normal levels, no detectable *RHO* transcript was found in NR2E3-null organoids at any time point (Figure 5A). Across D160 and D260, chromatin accessibility in the *RHO* coding sequences and *cis-*regulatory sites was greatly diminished in *NR2E3*-null rods compared with control rods (Figure 5, B and C). These putative regulatory sites were previously shown to be bound by CRX and NRL in ChIP sequencing (ChIP-Seq) experiments performed on human neural retina (28) (Figure 5D). D160 and D260 organoids were stained for rhodopsin expression, and a similar pattern was observed wherein no rhodopsin protein was found in the photoreceptors of mature NR2E3-null organoids (Figure 5, E–J).

### NR2E3 suppresses cone-specific gene expression in rods.

**Figure 6:**
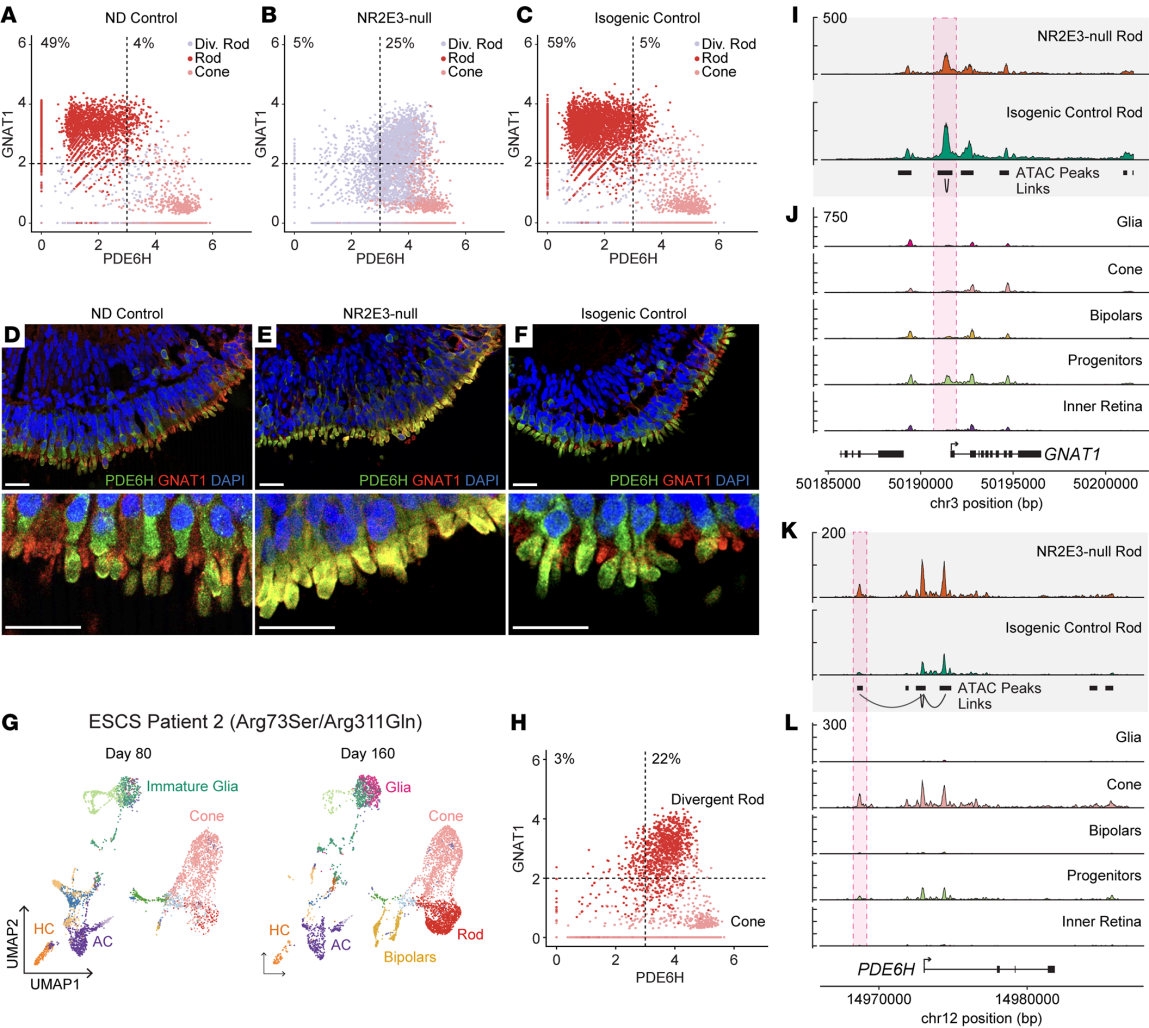
NR2E3 is required for repression of cone-specific phototransduction genes. (**A**) Cells of the photoreceptor lineage from the ND control line are plotted based on expression level of *PDE6H* (*x* axis) and *GNAT1* (*y* axis). Photoreceptor cells segregate by class with rods expressing *GNAT1* and cones expressing *PDE6H*. (**B**) Divergent rods coexpress *PDE6H* and *GNAT1* at high levels. No cells are observed to express only *GNAT1*, indicating lack of a normal rod population. *PDE6H*-expressing cone population is similar to controls. (**C**) The isogenic control line restores normal segregation of photoreceptor classes. (**D**) Segregation of expression of GNAT1 (red) and PDE6H (green) into rods and cones is observed in D260 ND control retinal organoids. (**E**) Photoreceptors from the NR2E3-null organoids exhibit colocalization of GNAT1 and PDE6H protein. (**F**) Segregation of expression is restored in isogenic control organoids. (**G**) Cells recovered from D80 and D160 organoids derived from a second ESCS patient were projected in 2D space using UMAP embeddings. Cells are shown grouped by cell type annotation derived from the first single-cell experiment (Figure 1). (**H**) *PDE6H* and *GNAT1* expression in rod and cone photoreceptors isolated from organoids from ESCS patient 2 (as shown in **G**). The proportion of cells coexpressing *GNAT1* and *PDE6H* is comparable to that in **B**. (**I**) ATAC coverage tracks for the rod cluster of organoids (D160 and D260 combined) are shown at the top for NR2E3-null and isogenic control samples. (**J**) Other cell type tracks show chromatin accessibility of the isogenic control sample. The *GNAT1* locus is shown. A peak linked to expression of *GNAT1* is shown boxed in red. This peak is accessible in both NR2E3-null and isogenic control rods. (**K**) The *PDE6H* locus is shown. NR2E3-null rods show accessibility at a peak normally accessibly only in cones (**L**). This peak is linked to expression of *PDE6H*. Scale bars: 50 μm.

To confirm that divergent rods genuinely coexpress both rod and cone genes, we subsetted the rod, cone, and divergent rod populations from the data set shown in Figure 2B and plotted cells along 2 axes for the canonical rod transducin component G protein subunit α transducin 1 (*GNAT1*) and the canonical cone phosphodiesterase 6H (*PDE6H*) (Figure 6, A–C). In both non-diseased (Figure 6A) and isogenic (Figure 6C) control samples, few photoreceptors coexpressed rod- and cone-specific genes (4% and 5%, respectively), with most cells exclusively expressing either *PDE6H* or *GNAT1*. However, divergent rods in the NR2E3-null organoids largely coexpressed both genes (25% of cells expressing either *GNAT1* or *PDE6H* coexpressed the markers) (Figure 6B). Such coexpression of *GNAT1* and *PDE6H* was further visualized at the protein level in mature organoids (Figure 6, D–F). Colocalization of GNAT1 and PDE6H protein in photoreceptor outer segments was never observed in the control lines (Figure 6, D and F) but was commonly observed in the NR2E3-null organoids (Figure 6E). These findings were confirmed by scRNA-Seq of late-stage (D260) organoids (Supplemental Figure 6).

To further confirm that divergent rod formation following NR2E3 loss of function was a generalized phenomenon and not genotype or patient specific, we differentiated an additional round of retinal organoids from an unrelated ESCS patient with a compound heterozygous genotype (c.219G>C [p.Arg73Ser]/c.932G>A [p.Arg311Gln]). Organoids from this line were sampled by scRNA-Seq at D80 and D160, and clusters were annotated via label transfer from the data set shown in Figure 1 and Figure 6G. Rod and cone populations were subsetted and plotted as in Figure 6, A–C and H. The majority of rod photoreceptors derived from the second ESCS patient organoids coexpressed GNAT1 and PDE6H, indicating that divergent rod formation occurs in different ESCS individuals with different causative genotypes.

GNAT1 is a canonically rod-specific component of the transducin complex. Accessibility at an ATAC peak near the promoter of *GNAT1* was found to be significantly correlated with expression of *GNAT1* (Figure 6I, red box). This peak was not accessible in non-rod retinal cells (Figure 6J). This rod-specific peak was observed in both NR2E3-null and isogenic control lines, indicating that loss of NR2E3 activity has no deleterious effect on rod-specific chromatin remodeling or transcription of *GNAT1* (as previously observed in Figure 6, B and E).

Loss of transcriptional repression of a cone-specific gene at the chromatin level following loss of NR2E3 was observed in the regulatory region surrounding *PDE6H* (Figure 6K). A cone-specific peak linked to expression of *PDE6H* was observed in NR2E3-null rods (Figure 6K, red box), indicating a failure to repress expression downstream of NR2E3 loss of this cone-specific member of the phosphodiesterase complex. The same peak in this presumptive enhancer of expression was observed in control cones, but not control rods (Figure 6L, red box). Together, these results show how NR2E3-null rods fail to suppress cone genes involved in phototransduction.

### Divergent rods are largely refractory to rescue by exogenous NR2E3 expression.

Expression and function of developmental transcription factors must be temporally controlled to enable cellular differentiation. Divergent rods form after expression of *NR2E3* is induced by *NRL* (Figure 4) (29). We next asked whether divergent rods observed in mature NR2E3-null organoids represent a state of paused differentiation, or a terminally differentiated cell type. Organoids were treated with adeno-associated virus (AAV) carrying either wild-type NR2E3 cDNA fused to T2A-GFP or GFP alone under a ubiquitous promoter (Supplemental Figure 6A). Treatment occurred at D130 of differentiation, after the deviation of divergent rods from the normal developmental trajectory (Figure 2). Successful transduction and expression of the transgene were confirmed by GFP expression (Supplemental Figure 6). Thirty days after treatment (D160), organoids were dissociated and processed for scRNA-Seq (Supplemental Figure 6B). Cell types were annotated (Supplemental Figure 6C), and cells expressing the AAV-derived NR2E3-T2A-GFP (or GFP alone) construct were positively identified via mapping to a custom reference genome containing GFP sequence (Supplemental Figure 6D). Transduction and restoration of NR2E3 protein expression in transduced cells were confirmed by immunohistochemistry (Supplemental Figure 6, E and F).

Rod photoreceptors were binned by transgene expression, and differential expression analysis was performed between transduced and untransduced populations within the AAV-NR2E3-EGFP–treated sample (Supplemental Figure 6). Other than expression of the transgene itself, several rod-specific phototransduction genes were upregulated in rods transduced with AAV-NR2E3-EGFP compared with untransduced rods. Next, expression of *PDE6H* and *GNAT1* was visualized in each sample (Supplemental Figure 6, H and I). Coexpression of these markers was shown above to exclusively mark divergent rods in the patient organoids (Figure 6). In untransduced rod photoreceptors, all cells exhibited expression of the cone-specific marker *PDE*6*H* (Supplemental Figure 6H). A small proportion of NR2E3-T2A-GFP–expressing rod photoreceptors showed absent or diminished *PDE6H* expression and higher *GNAT1* expression (Supplemental Figure 6I), resembling the profile of normal rod photoreceptors. Twenty-two percent (46/207) of transduced GNAT1^+^ rods displayed suppression of *PDE6H*, while 5% (39/727) of untransduced GNAT1^+^ rods displayed *PDE6H* suppression. The expression pattern of GFP-only–expressing rods resembled that of untransduced cells (Supplemental Figure 6J). Notably, most transduced divergent rods did not show appreciable downregulation of *PDE6H* and had an expression pattern that resembled that of untreated divergent rods (Supplemental Figure 6I). This may indicate that while a minority of divergent rods can suppress *PDE6H* expression following the reintroduction of NR2E3, the majority are refractory by 130 days of differentiation.

### NR2E3-null divergent rods are transcriptionally distinct from NRL-null cods.

**Figure 7:**
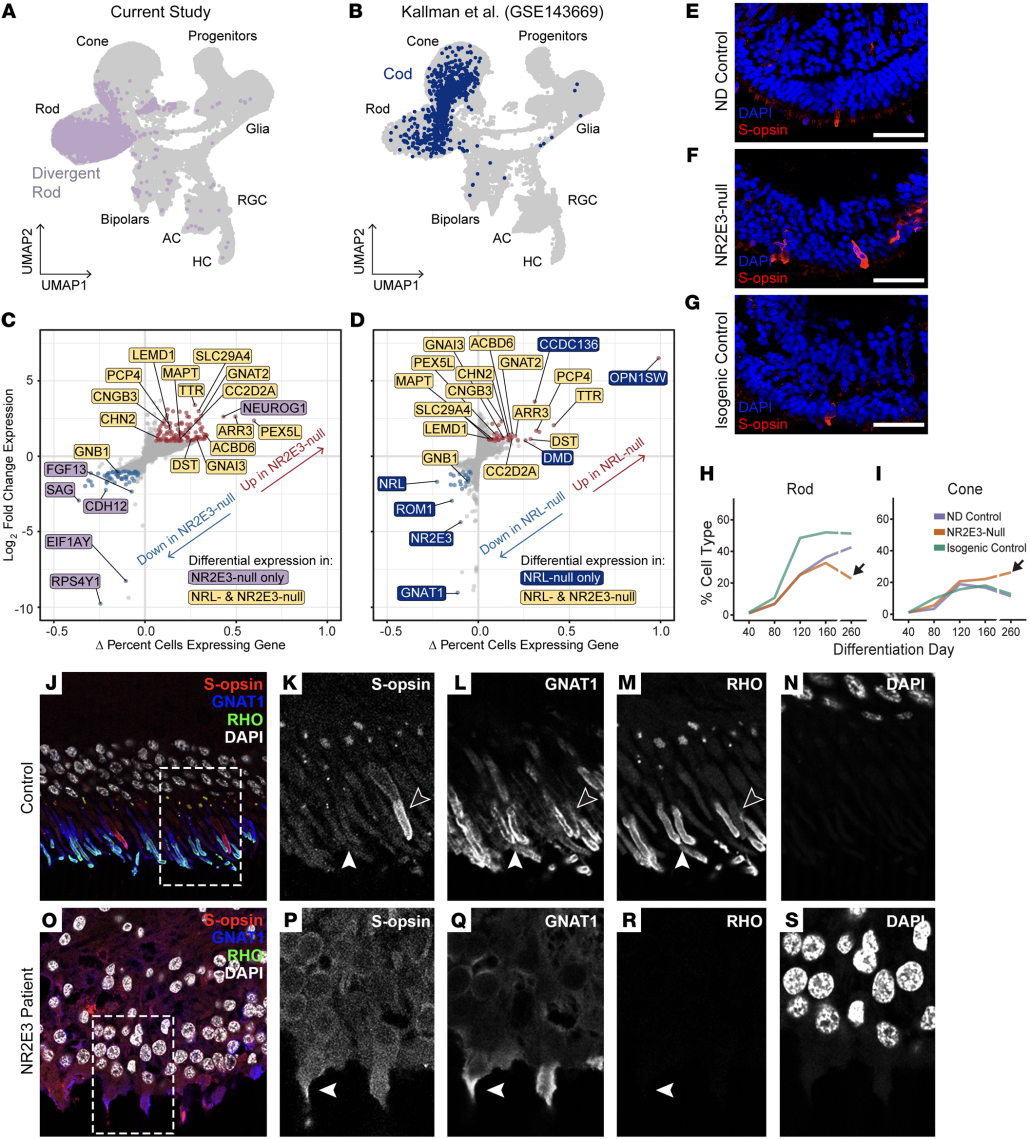
Divergent rod fate in the context of ESCS. (**A** and **B**) D40–D260 data from the photoreceptor lineage of the current study integrated with the same cell types from Kallman et al. (31). Cells are shown split by study and projected in 2D space using UMAP embeddings. Divergent rods are colored lavender, and NRL-null cods are colored blue. (**C** and **D**) Differential expression analysis between pathological and normal rods from each study. Genes in yellow are significantly dysregulated in both NRL- and NR2E3-null cells compared with control rods. Genes in lavender (**C**) are dysregulated exclusively in divergent rods. Genes in blue (**D**) are dysregulated exclusively in NRL-null cods. (**E**–**G**) D260 retinal organoids from the current study stained for S-opsin. NR2E3-null organoids display a modest increase in the proportion of S-opsin–expressing cells. (**H** and **I**) Between D160 and D260 the rod proportion of NR2E3-null organoids decreases while the cone proportion increases. The opposite trend is observed in controls. (**J**) Staining of control postmortem donor retina shows rare short-wavelength cones (S-opsin), and colocalization of rhodopsin and GNAT1 in rods. (**K**–**N**) Cropped image from **J** showing S-cone (black arrowheads) and rods (white arrowheads). (**O**) In an NR2E3 disease donor retina, no rhodopsin staining is observed, and colocalization of S-opsin and GNAT1 is present. (**P**–**S**) Cropped image from **O** showing photoreceptor coexpressing S-opsin and GNAT1 (white arrowheads). Scale bars: 50 μm.

ESCS is most often caused by mutations in *NR2E3* (30). However, rare cases of ESCS are known to be caused by mutations in the genetically upstream rod photoreceptor–specific transcription factor gene *NRL* (31). A previous study of human retinal organoids lacking NRL activity described the presence of hybrid cone/rod photoreceptor cells termed “cods,” described earlier in the Nrl^–/–^ mouse (11, 31). As NRL is the inducer of NR2E3 expression in normal rod development (23), cods lack NR2E3 expression (31). We therefore asked how divergent rods differed at the transcriptome level from cods to describe the specific contribution of NR2E3 to rod development genetically and temporally downstream of NRL. We generated scRNA-Seq profiles of D260 organoids (Supplemental Figure 7). Data were integrated with those of Kallman et al. (31), and the divergent rod and cod populations were annotated (see Methods) and highlighted (Figure 7, A and B), showing that cods cluster more closely to normal cones, while divergent rods cluster more closely to normal rods. Differential expression analysis was performed between divergent rods and normal rods from the current study and between cods and normal rods from the Kallman et al. study to remove confounding effects introduced by differences in the differentiation protocol used by each study. The fold change and percentage of cells expressing each gene are plotted for both analyses (Figure 7, C and D). While cods and divergent rods dysregulated expression of many common genes (Figure 7, C and D, yellow labels), several genes were dysregulated only in cods or divergent rods, respectively (Figure 7, C and D, lavender and blue labels). This indicates that NR2E3 and NRL regulate partially exclusive subsets of genes essential to rod development and function.

In addition to a lack of normal rod photoreceptor function, ESCS is characterized by an exaggerated retinal response to short-wavelength light. Previous studies in NRL-null organoids (31) and postmortem examinations of NR2E3-associated ESCS eyes (12) have shown an increased number of S-opsin–expressing cells in the ESCS photoreceptor cell layer. However, there are drastic differences in the observed magnitude of fate conversion of rods to S-cones in NRL-null animal models versus NR2E3-null models and patient observations. NRL-null organoids exhibited a complete conversion, with seemingly all rods becoming OPN1SW-expressing cods early in development. Milam et al. (12) showed that in NR2E3 patient retinas, the number of cone photoreceptors was only increased 2-fold, with the vast majority of those expressing OPN1SW. We observed only a modest increase in S-opsin–expressing cells in NR2E3-null organoids (Figure 7, E–G) and no change in the amount of ML-opsin–expressing cells (Supplemental Figure 8, G–I). We integrated our scRNA-Seq data from D40 to D260 of differentiation to understand the late fate of divergent rods. By D260, no early or intermediate progenitors existed (Supplemental Figure 8), and the proportion of other major cell types was equivalent between lines (Supplemental Figure 8). Examining the rod cluster, we observed a decrease in the proportion of rods between D160 and D260 only in the NR2E3-null line (Figure 7H). We observed the opposite trend in the cone cluster (Figure 7I), where the proportion of cones in the NR2E3-null organoids increased between D160 and D260, decreasing in the control lines. This was not the result of proliferation of progenitor cells or death of a certain population of cell, because there was no difference in Ki67 (proliferation marker) or cleaved PARP (apoptosis marker) at D40 or D260 (Supplemental Figure 8, E and F). These data suggest that a subset of divergent rods may give rise to late-born cone photoreceptors in NR2E3-null retinal organoids.

### NR2E3 is not required for rod specification in an NR2E3 patient eye.

The composition of the photoreceptor mosaic of patients with NR2E3 disease has previously been largely inferred from electroretinogram testing and clinical imaging (30, 32–34). Postmortem studies of patient eyes affected by NR2E3 disease have been limited to broad cone cytoplasmic markers, structural electron microscopy, and opsin staining (12, 35, 36). These studies have shown that an increased number of short-wavelength opsin-expressing cells exist in NR2E3 patient retina and that no rhodopsin-expressing cells are present. Further, the retinal architecture and opsin localization become disordered in comparison with control samples. However, these studies have not addressed whether rod photoreceptor–fated cells exist in NR2E3 patient retinas. We next asked whether the divergent rod photoreceptors we observe emerging in NR2E3 patient organoids form and persist throughout life in the NR2E3 patient retina.

We stained postmortem central retina tissue from a previously described patient with NR2E3-associated Goldmann-Favre syndrome (35) for GNAT1, rhodopsin, and S-opsin (Figure 7, O–S). In control retina, GNAT1 and rhodopsin colocalized in rod photoreceptors, while S-opsin was expressed in rare short-wavelength cones (Figure 7, J–N). In the NR2E3 patient retina, we observed strong expression of GNAT1, indicating rod commitment and persistence similar to what we observed in retinal organoids (Figure 7, O–S). As in previous studies (12, 35), we observed no rhodopsin expression within GNAT1^+^ cells. However, we did observe colocalization of GNAT1 and S-opsin, implying that divergent rods may express the cone opsin during life.

## Discussion

Much of the cellular biology underlying human retinal disease must be inferred from clinical imaging studies, animal models, or postmortem case studies. This is primarily due to the relative inaccessibility of retina in living patients for research studies compared with easily biopsied tissues such as blood. As such, the precise downstream effects of loss of developmental genes in the human retina are largely unknown. In this study, we used patient-derived iPSC-based modeling to capture developing human retinal cells with a clinically relevant mutation. We show that sampling developing organoids at multiple time points using scRNA-Seq allows precise identification of the immediate consequences of NR2E3 loss following rod photoreceptor commitment. We demonstrate that NR2E3 loss in human retinal cells specifically causes misregulation of several rod- and cone-specific phototransduction genes, including loss of *RHO* expression. Using joint multimodal single-cell RNA and ATAC sequencing, we identify the putative *cis*-regulatory sites of the misexpressed phototransduction genes and show how loss of NR2E3 alters chromatin accessibility in both activating and repressive roles. Interestingly, loss of Nr2e3 in mice does not have the same effect. Specifically, while mouse Nr2e3 is sufficient to induce Rho expression in an Nrl-null background (23), Nr2e3 is not required for Rho expression in mice (16). Together, our findings show how an in vitro model of human retinal development can be used to parse the species-specific roles of a core developmental transcription factor with implications for inherited retinal disease.

### Characterization of divergent rods.

In this study, we identified a population of rod photoreceptor cells unique to the NR2E3-null disease state. NR2E3-null retinal progenitors differentiate normally until a late time point, after the induction of NRL specifies rod photoreceptors. Because of this, prior to D120, divergent rod photoreceptor cells are indistinguishable from normal rods and earlier progenitors. These data indicate that NR2E3 loss in human cells does not delay progenitor commitment nor shunt presumptive rods into a cone fate during early development, counter to hypotheses based on clinical imaging and mouse models. Instead, the primary defect of NR2E3-null divergent rods seems to involve late genes involved in maturation and rod function, rather than commitment. Notably, this is dissimilar to the defect observed in NRL-null human retinal organoids (31), wherein cods appeared to be in a state of arrested commitment, expressing markers of T3 photoreceptor progenitors such as *FABP7* and *ISOC1* (described in human fetal retina and retinal organoids) (21).

### NR2E3/NRL cooperation in rod development.

Rhodopsin (*RHO*) is the light-sensitive opsin that allows rod photoreceptors to detect and trigger the signals required to enable vision in dim light. Rhodopsin is expressed exclusively in rods following early differentiation from uncommitted photoreceptor progenitors. Understanding how and when upstream factors control *RHO* expression is essential to addressing photoreceptor dysfunction in the disease state. Expression of *RHO* is thought to be under the control of the rod-specific transcription factors NRL and NR2E3; however, several lines of evidence imply major regulatory differences between human and murine *RHO* regulation. As indicated above, NR2E3-deficient mice express rhodopsin in rod photoreceptors, as do NRL-deficient animals expressing NR2E3 under other rod-specific promoters (23), which suggests a compensatory relationship between NR2E3 and NRL wherein either is sufficient to drive rhodopsin expression in mice. In human retinal organoids lacking NRL, neither NRL nor NR2E3 is functional, and rhodopsin is not expressed (31). Here, we show that even in rod photoreceptors with normal levels of functional NRL, loss of NR2E3 is sufficient to prevent rhodopsin expression.

The rather differentiated state of divergent rods raises the question of their functionality in terms of light sensitivity, synapse formation, and other known photoreceptor functions. The lack of rhodopsin expression implies that divergent rods would be insensitive to light. However, their expression of other phototransduction genes indicates that they may retain some ability to conduct electrochemical signal. Divergent rod photoreceptors appear to be positioned in the appropriate location in the laminated neural retina both in donor samples and in organoids. This implies that they may have synaptic connections to the bipolar cells of the inner retina. Ultimately, the function of these cells can be tested in vitro during electrophysiological assays such as multielectrode array, which others have used to test retinal organoid light sensitivity (37).

### Differentiation potential of divergent rod photoreceptors.

NR2E3 is known to act in terminally differentiated rod photoreceptors and is required in adulthood to enforce expression of rod function genes and repress expression of cone function genes. We tested whether supplementation of wild-type NR2E3 into divergent rod cells after the normal temporal window on NR2E3 induction would be sufficient to revert cells to a normal pattern of gene expression. If so, divergent rods could be thought to represent an incompletely differentiated photoreceptor. However, we found that most divergent rods do not revert to normal rod phenotype within 30 days following transduction with an AAV carrying wild-type *NR2E3*. This may indicate that the divergent rod state represents a terminally differentiated alternate cell fate undertaken by developing rods in the absence of NR2E3. In this case, a self-enforcing gene regulatory network supporting expression of divergent rod genes may be resistant to reprogramming by NR2E3 once established. Confirmation of this hypothesis would require future molecular studies of the chromatin state of divergent rods and testing of NR2E3’s ability to alter this landscape in mature divergent rods.

### Supernumerary S-cones in NR2E3-null retina.

ESCS is characterized by an increased retinal sensitivity to short-wavelength light, mediated by S-cones, and a loss of sensitivity to dim light, mediated by rod photoreceptors. Due to the known role of NR2E3 in rod photoreceptor fate specification, supernumerary S-cone formation in lieu of rod formation has been proposed as the explanation for cone hypersensitivity. Postmortem study of ESCS patient retinas revealed a slight increase in the proportion of cones, approximately 2-fold greater than normal, with most of these expressing S-opsin (12). This is similar to what we observed in the current study (Figure 7) but is contrary to what is observed following loss of *NRL* (31). Interestingly, the increase in S-cone number in our study was not readily apparent until 260 days of differentiation, which is quite late in development. With an increased proportion of S-cones, we observed a concomitant decrease in the proportion of divergent rods. Since there was no increase in the rate of apoptosis to suggest divergent rod cell death, we hypothesized that under prolonged cell culture divergent rods may transdifferentiate into S-cones. This could occur by either direct conversion or reversion to an earlier developmental state followed by progression down a blue cone developmental pathway. To evaluate this hypothesis, further experimentation will be required to determine whether a subpopulation of NRL-expressing divergent rods are able to silence rod gene expression and revert to a bona fide blue cone cell state.

### Conclusion.

In summary, we demonstrate the power of a combined patient-derived induced pluripotent stem cell, CRISPR-based genome editing, and single-cell sequencing strategy to elucidate the role of the transcription factor NR2E3 in human retinal development and disease. We demonstrate that loss of the transcription factor, which is essential for rod photoreceptor function, has a very different outcome in humans as compared with rodents. These differences are critical for understanding how the human retina develops.

### Limitations of study.

Modeling retinal development with patient-derived stem cells and retinal organoids offers a unique and clinically relevant view of the molecular changes downstream of transcription factor loss. However, the organoid model is subject to several limitations. While we attempted to infer functionality of divergent rods based on expression of key phototransduction genes at the protein and transcript level, further work will be needed to determine what, if any, electrical response to light is present in such cells. Rod and cone photoreceptor morphology differs greatly in vivo. The relative immaturity of structural aspects of organoid-derived photoreceptors such as outer segments limited our analysis of this phenotype. Additionally, the retinal organoid model is limited by lack of adjacent cell types that may modulate disease phenotype expression, namely vascular cells and the retinal pigment epithelium that serves to support photoreceptor physiology in vivo and immune cells such as microglia that may play a role in pruning divergent rod cells in vivo. However, histological examination of a postmortem NR2E3 disease eye shows the presence of photoreceptors expressing rod-specific proteins in the ninth decade of life. This finding suggests that divergent rods form in vivo and persist late into adulthood. Finally, while we observed an increase in the proportion of S-cones concomitant with a decrease in divergent rod proportion between D160 and D260 with no obvious changes to proliferation or cell death, further work will be required to lineage-trace developing divergent rods and determine their fate in developing organoids.

## Methods

Further information can be found in Supplemental Methods.

### Sex as a biological variable.

iPSC lines from both male and female donors were used in this study.

### Patient-derived iPSC generation and validation.

This study was approved by the Institutional Review Board of the University of Iowa (project approval 200202022) and adhered to the tenets set forth in the Declaration of Helsinki. Patient iPSCs were generated from an individual with no disease and 2 individuals with molecularly confirmed ESCS (20, 38). The disease-causing mutation in NR2E3, c.119-2A>C, was corrected via CRISPR-mediated homology-dependent repair in patient-derived iPSCs as described previously (20).

### Retinal organoid differentiation.

Retinal differentiation was performed as described previously with minor modifications (20, 39). Briefly, iPSCs were cultured on laminin 521–coated plates in E8 medium. Embryoid bodies (EBs) were lifted with ReLeSR (STEMCELL Technologies) and transitioned from E8 to neural induction medium (NIM) over a 4-day period. On day 6, NIM was supplemented with 1.5 nM BMP4 (R&D Systems). On day 7, EBs were adhered to Matrigel-coated plates (Corning). BMP4 was gradually transitioned out of the NIM over 7 days. On day 16, the medium was changed to retinal differentiation medium (RDM). On day 25–30 the entire EB outgrowth was mechanically lifted and transferred to ultra-low-attachment flasks in 3D-RDM (RDM plus 10% FBS; Thermo Fisher Scientific), 100 μM taurine (Sigma-Aldrich), 1:1,000 chemically defined lipid concentrate (Thermo Fisher Scientific), and 1 μM all-*trans* retinoic acid (until day 100; Sigma-Aldrich). The cells were fed 3 times per week with 3D-RDM until harvest.

### Immunocytochemistry.

Organoids were fixed with 4% paraformaldehyde for 30–60 minutes at room temperature and equilibrated to 15% sucrose in PBS, followed by 30% sucrose. Organoids were cryopreserved in 50:50 solution of 30% sucrose-PBS to tissue freezing medium (Electron Microscopy Sciences) and cryosectioned (15 μm). Sections were blocked with 5% normal donkey serum, 3% BSA, and 0.1% Triton X and stained overnight with the primary antibodies listed in Supplemental Table 1. Secondary antibodies (Supplemental Table 1) were incubated for 1 hour, and cell nuclei were counterstained using DAPI (Thermo Fisher Scientific, catalog 62248). Human donor sections were fixed in a mixture of 4% paraformaldehyde and 0.5% glutaraldehyde made in 0.1 M phosphate buffer, pH 7.3. After 1 month in fixative, the globes were transferred and stored in 2% paraformaldehyde prepared in the same buffer as previously described (35). Human donor sections were blocked in 1 mg/mL BSA for 15 minutes at room temperature, incubated with primary antibody for 1 hour at room temperature, and incubated with secondary antibody and DAPI for 30 minutes at room temperature. Images were acquired using a Leica TCS SPE upright confocal microscope system (Leica Microsystems).

### Organoid dissociation for scRNA-Seq.

Samples were collected at differentiation days 40, 80, 120, 160, and 260 and processed for single-cell gene expression profiling. Approximately 10 organoids displaying morphology of successful retinal differentiation were selected for each line. Organoids were settled by gravity, and culture medium was removed. Organoids were dissociated in a 300 μL solution of 20 U/mL papain (Worthington) and 120 U/mL DNase (Worthington) in Earle’s balanced salt solution (Worthington). Samples were incubated at 37°C with continuous shaking (500 rpm) and were triturated with a pipette every 15 minutes until all organoids were completely dissociated (approximately 1 hour). Cells were pelleted at 500*g* for 5 minutes and resuspended in 8 μg/mL recombinant albumin (New England Biolabs) in dPBS–/–. Cells were passed through a 70 μm filter to encapsulation with the Chromium Controller instrument (10X Genomics). Approximately 8,000 cells were targeted for encapsulation per sample.

### Nucleus isolation for single-nucleus multimodal sequencing.

Nuclei were isolated from D160 and D260 organoids cultured in an independent batch from those used in the scRNA-Seq study above. Nuclei were isolated following a protocol based on 10X Genomics’s demonstrated protocol CG000366. Briefly, approximately 10 organoids were selected as described above. Medium was aspirated, and 500 μL of chilled 0.1× Lysis Buffer (Tris-HCl, NaCl, MgCl_2_, Tween-20, NP-40, digitonin, BSA, DTT, RNase inhibitor) was added to each sample. Samples were homogenized with 15 strokes of a sterile pestle on ice and incubated for 5 minutes on ice. Samples were mixed with a P1000 pipette 10 times on ice and incubated for 8.5 minutes on ice. Five hundred microliters of wash buffer (Tris-HCl, NaCl, MgCl_2_, Tween-20, BSA, DTT, RNase inhibitor) was added, and lysed cells were mixed 5 times with pipetting. Cells were pelleted at 500*g* for 5 minutes in a 4°C centrifuge and resuspended in 500 μL chilled wash buffer. This process was repeated for 3 total washes. Nuclei were resuspended in diluted nuclei buffer (Nuclei Buffer [10X Genomics], DTT, RNase inhibitor) and passed through a 70 μm strainer. Nuclei were counted on a hemocytometer with DAPI to visualize intact nuclei. Approximately 9,000 nuclei were targeted for encapsulation per sample using the Chromium X instrument (10X Genomics).

### Single-cell gene expression library preparation and sequencing.

Single cells were partitioned and barcoded with the Chromium Controller instrument (10X Genomics) and Single Cell 3′ Reagent (v3.1 chemistry) kit (10X Genomics) according to the manufacturer’s specifications with no modification (Rev. C). Final libraries were quantified using the Qubit dsDNA HS Assay Kit (Life Technologies) and diluted to 3 ng/μL in buffer EB (Qiagen). Library quality was confirmed using the Bioanalyzer High Sensitivity DNA Assay (Agilent) before sequencing.

### Single-nucleus multimodal library preparation and sequencing.

Nuclei were processed following the 10X Genomics Chromium Next GEM Single Cell Multiome ATAC + Gene Expression (Rev. E) without modification. Final libraries were quantified using the Qubit dsDNA HS Assay Kit and diluted to 3 ng/μL. Library quality was checked using the Bioanalyzer or Tapestation (Agilent).

### Single-cell gene expression data integration and processing.

scRNA libraries were pooled and sequenced using the NovaSeq 6000 instrument (Illumina) generating 100 bp paired end reads. Sequencing was performed by the Genomics Division of the Iowa Institute of Human Genetics. FASTQ files were generated from base calls with bcl2fastq software (Illumina), and reads were mapped to the pre-built GRCh38 reference (refdata-gex-GRCh38-2020-A) with Cell Ranger v7.0.0 (10X Genomics) using the “count” function with the following parameters: --expect-cells = 8,000 --localcores = 56. Only cells passing the default Cell Ranger call were analyzed further. Differentiation day 40–160 samples were integrated using canonical correlation analysis (CCA) in Seurat v4.0.3 (40). Only cells with between 1,000 and 7,000 unique genes (features) were included in the analysis. Only cells with less than 10% of reads mapping to mtDNA-encoded genes and less than 20% of reading mapping to ribosomal genes were included. Counts data were normalized using the NormalizeData function (Seurat) with the following parameters: normalization.method = LogNormalize, scale.factor = 10,000. Two thousand variable features were identified with the FindVariableFeatures function using the vst selection method. Integration anchors were identified with the FindIntegrationAnchors function using 25 dimensions. An assay “Integrated” was generated for the 2,000 variable features with the IntegrateData function using 25 dimensions. The integrated data were then used in principal component analysis (PCA).

### Single-nucleus multimodal data integration and processing.

Single-nucleus multimodal libraries were sequenced using the NovaSeq 6000 instrument (Illumina). Sequencing was performed by the Genomics Division of the Iowa Institute of Human Genetics. FASTQ files were generated from base calls with bcl2fastq software (Illumina). Reads were mapped to the pre-built GRCh38 reference (GRCh38-2020-A-2.0.0, 10X Genomics) using Cell Ranger ARC (v2.0.0, 10X Genomics) with default parameters. Resulting cell-by-peak and cell-by-gene matrices (ATAC and gene expression assays, respectively) from the 4 samples were integrated separately using latent semantic indexing (ATAC) and CCA (gene expression).

### Dimensionality reduction with UMAP and cell type annotation.

Thirty principal components were identified out of the D40–D160 integrated data set described above using the RunPCA function (Seurat) (40). Uniform manifold approximation and projection (UMAP) was performed with the RunUMAP function using 25 principal components. Cells were annotated with the FindTransferAnchors and TransferData functions using 30 principal components and data from ref. 21 as a reference. The photoreceptor cluster was manually refined to rod and cone photoreceptor cell classes based on expression of several canonical marker genes (e.g., *ARR3*, *GNGT1*, *RCVRN*, etc.). Plots were generated using scCustomize (v2.0.1) (41).

### Dimensionality reduction of multimodal data with WNN-UMAP and cell type annotation.

Only cells passing both gene expression and ATAC assay quality control metrics were used in downstream analysis. A weighted nearest neighbor (WNN) graph was constructed based on a weighted combination of either sequencing modality (gene expression [GEX] and ATAC). From the GEX data, dimensions 1–30 of the PCA reduction from CCA were used. From the ATAC data, dimensions 2–50 of the latent semantic indexing were used. UMAP was performed on the resulting WNN graph. Clusters were identified based on the weighted shared nearest neighbor graph using the SLM algorithm with a resolution of 0.5 using the FindClusters function in Seurat. Cluster identity (i.e., cell type) was assigned based on expression (from the RNA assay) of cell type–specific marker genes.

### Dimensionality reduction and cell type annotation with PHATE.

Using the annotations described above from the differentiation day 40–160 data set, cells within the photoreceptor developmental lineage were identified (i.e., Progenitors, T1, T3, Rod, and Cone). SCTransform (42) was performed on this subset to scale and normalize the counts. PHATE (25) was run on the SCT data using the following parameters: knn = 6, decay = 50, t = 100. Next, clustering was performed on the PHATE-derived embedding. Neighbors were identified with the FindNeighbors function (Seurat) using dimensions 1 and 2 of the PHATE reduction. Clusters were identified using the FindClusters function (Seurat) with the following parameters: resolution = 0.5, algorithm = 3. The resulting clusters were combined and annotated based on time point and cell type annotation from PCA-based clustering in the previous section.

### Trajectory construction with Slingshot.

Slingshot (26) was used to infer trajectories through the PHATE reduction of the photoreceptor lineage and assign pseudotime values to cells based on principal curves through identified trajectories. The Slingshot function was run taking PHATE embeddings and PHATE-derived cluster labels as “input.” The starting (Progenitor) and end (Cone, Rod, Divergent Rod) clusters were given, and the following parameters were used: extend = *n*, stretch = 0.1, thresh = 0.1, approx_points = 150. Three trajectories were identified. Curves were drawn based on these trajectories from which pseudotime values were derived and given to each cell.

### Differential gene expression analysis.

The FindMarkers function (Seurat) identified differentially expressed genes between divergent rods and rods and between divergent rods and cones. Only features (genes) with a mean count of at least 1 across all cells in the photoreceptor lineage (Progenitors, T1, T3, Rod, Cone) were used. Genes with a log_2_(fold change) of at least 1 and an adjusted *P* value below 0.05 were considered significantly differentially expressed. These genes were used to identify enriched pathways using the Ingenuity Pathway Analysis application.

### Transcription factor binding motif enrichment analysis.

ATAC peaks to be used in motif enrichment analysis were first called using MACS2 (43, 44) with the CallPeaks function in Signac. Cells were grouped by cell type as described above. To identify transcription factor binding motifs enriched in differentially accessible regions in rods and divergent rods, the FindMarkers function was used with the following parameters: subset.ident = Rod, only.pos = FALSE, test.use = LR, latent.vars = nCount_macs2_peaks. Differentially accessible regions were filtered for *P* less than 0.05. Enriched motifs were identified in differentially accessible regions using the FindMotifs function, with a set of 50,000 GC-matched peaks accessible in the same cell type used as background. Enrichment over background and significance of motifs were plotted, highlighting the motifs of interest (i.e., NRL and NR2E3).

### Retina ChIP-Seq visualization.

Human ChIP sequencing data (28) were accessed from Gene Expression Omnibus (GEO) GSE137311 (specifically samples GSM4075125 [NRL] and GSM4075107 [CRX]). BigWig files were downloaded and visualized alongside organoid chromatin accessibility data using the CoveragePlot function in Signac.

### Peak-to-gene linkage analysis.

Peak-to-gene linkages were identified using the strategy described in SHARE-seq (45) and implemented with Seurat/Signac (46). Links were computed between genes known to be involved in phototransduction (i.e., *GRK*, *GRK7*, *RCVRN*, *RHO*, *SAG*, *GNAT1*, *GNAT2*, *GNB1*, *GNGT1*, *RGS9*, *PDE6A*, *PDE6G*, *PDE6B*, *PDE6H*, *GUCY2F*, *GUCY2D*, *GUCA1A*, *GUCA1B*, *GUCA1C*, *SLC24A1*, *CALML6*, *CALML5*, *CALM1*, *CALM2*, *CALM3*, *CALML3*, *CALML4*, *CNGB1*, *CNGA1*) and proximal ATAC peaks as identified by MACS2 (above). Links were calculated using the LinkPeaks function (Signac) with the following custom parameter: distance = 10e+05. Links were visualized using the CoveragePlot function (Signac).

### Integration and comparison with previously published scRNA-Seq data.

Single-cell sequencing data from NRL-null and control retinal organoids were accessed from GSE143669 (31). Data from the current study (D40–D260) and data from Kallman et al. (31) were integrated using CCA. Counts were normalized using LogNormalize in the NormalizeData function in Seurat, and 2,000 variable features were identified using the vst selection method. Integration anchors were identified using the first 25 principal components. UMAP was run using the first 25 principal dimensions of the integrated object. Cell types were annotated using the FindTransferAnchors function using the D40–D260 data set from the current study as the reference. A rod and cone gene module score was computed using the AddModuleScore function (Seurat). The rod module consisted of the following genes: *ROM1*, *PDE6G*, *SAG*, *NRL*, *NR2E3*, *CNGB1*, *GNAT1*. The cone module score consisted of the following genes: *ARR3*, *CNGB3*, *GNAT2*, *GNB3*, *GNGT2*, *GUCA1A*, *PDE6C*, *PDE6H*.

For this analysis (shown in Figure 7), divergent rods were defined using the following criteria: (a) from the current study; (b) from D160 or D260 time point; (c) predicted annotation as rod from the above label transfer; (d) from the NR2E3-null line. Cods (i.e., NRL-null hybrid photoreceptors) were defined using the following criteria: (a) from the Kallman et al. study (31); (b) rod gene module score less than 2; (c) cone gene module score less than 2; (d) OPN1SW log-normalized counts greater than 0.5; (e) from the NRL-null line (NRL_L75P); (f) predicted annotation as either rod, cone, T1, T3, or PRC/photoreceptor from the above label transfer.

Differentially expressed genes between cods and rods from the Kallman et al. data set (31) and between divergent rods and rods from the current data set were identified using the FindMarkers function in Seurat. To generate the plots in Figure 7, C and D, the gene list was filtered to only genes expressed in at least 10% of rods (either normal or abnormal) in both data sets. Differential expression was defined as log_2_(fold change) greater than 1 within this subset.

### Statistics.

Differential accessibility for motif enrichment analysis was calculated using a likelihood ratio test with adjusted *P* value (Bonferroni’s correction) of less than 0.05. Differential expression in Figure 4 was calculated using Wilcoxon’s rank sum test with adjusted *P* value (Bonferroni’s correction) of less than 0.05.

### Study approval.

This study was approved by the institutional review boards of the University of Iowa and adhered to the tenets set forth in the Declaration of Helsinki. Written informed consent was obtained from all subjects prior to participation.

### Data availability.

Raw and processed data from single-cell experiments are available on GEO under accession number GSE236197. Processed data are also available for interactive exploration online on the Spectacle platform (47) (https://singlecell-eye.org). Code used to process and analyze the sequencing data is available on GitHub (https://github.com/nkmullin/nr2e3_organoid_2023). Values for data shown in graphs can be found in the Supporting Data Values file.

## Author contributions

NKM, LRB, and BAT conceptualized the study. NKM, LRB, and APV devised methodology. NKM, LRB, and APV performed formal analysis. NKM, LRB, LPL, and ATW performed investigation. VLB, EMS, and BAT provided resources. NKM and LRB wrote the original draft. APV, LPL, ATW, RFM, EMS, BAT, and VLB reviewed and edited the manuscript. NKM, APV, RFM, EMS, and BAT acquired funding. RFM and BAT supervised the study.

## Supplementary Material

Supplemental data

Supporting data values

## Version 1 04/23/2024

In-Press Preview

## Version 2 06/03/2024

Electronic publication
