# Supplementary material for: NR2E3 loss disrupts photoreceptor cell maturation and fate in human organoid models of retinal development
Source: J Clin Invest. 2024 Apr 23;134(11):e173892. doi: 10.1172/JCI173892 (PMC11142732; doi:10.1172/JCI173892)
Supplement: Supplemental data [file jci-134-173892-s171.pdf]

Supplemental Methods

iPSC characterization. Following generation and clonal expansion, iPSCs were karyotyped in metaphase by the Shivanand R. Patil Cytogenetics and Molecular Laboratory at the University of Iowa using Leica Microsystems metaphase scanning platform and CytoVision version 7.7 software. Cells were grown *in vitro* and arrested at metaphase with colcemid. Chromosomes were stained by the G-banding method, counted, and structurally evaluated for the presence or absence of detectable rearrangements. At least 20 cells were analyzed for each iPSC line. The complete NR2E3 locus was a sequenced using long reads (Oxford Nanopore Technologies) of an 8kb amplicon to confirm the lack of any potentially damaging variants.

AAV transduction. Approximately 10 D130 NR2E3-null organoids (c.119-2A>C/c.119-2A>C) were transduced with adeno-associated virus carrying GFP ($2x10^{12}$ GC/mL) or NR2E3-T2A-GFP ($3x10^{12}$ GC/mL) (VectorBuilder, VB230812-1083xdt). Fresh media was added the next day and 5 days later the transduction was repeated. At D160 (i.e., D30 post-transduction), we assayed organoids by scRNAseq and IHC as described previously. Organoids for the scRNAseq experiment were transduced with plasmid packaged with AAV1.

Mapping of sequencing reads from scRNAseq of AAV-transduced organoids. Custom reference genomes were built to allow mapping of EGFP and associated 3'UTR regions originating from AAV cargo. For AAV-NR2E3-T2A-EGFP transduced and untransduced samples, reads were mapped to a reference genome containing T2A-EGFP-BGHpolyA sequence. For AAV-EGFP transduced samples, reads were mapped to a reference genome containing EGFP-WPRE-BGHpolyA sequence. Data from all samples was integrated using CCA. The top 2000 variable features as determined by vst method were used. For AAV-transduced samples, the transgene feature was not included as a variable feature.

Supplemental Figures

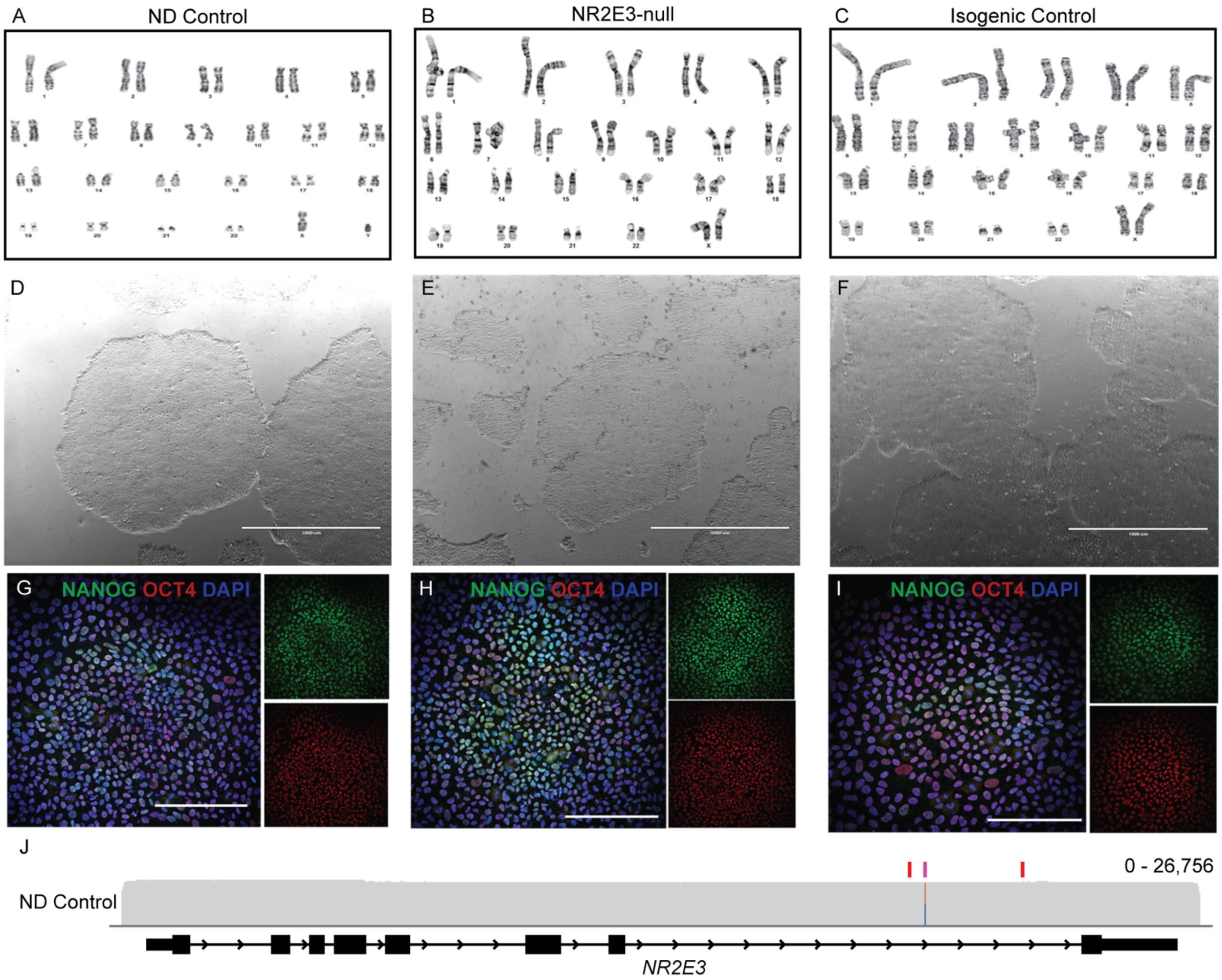

Figure S1. Characterization of iPSC lines. A-C) All iPSC lines displayed a normal karyotype by G-banding analysis. **D-F)** Patient and control lines displayed typical iPSC colony morphology throughout culture. **G-I)** Undifferentiated iPSC lines express markers of pluripotency including Nanog and OCT4. **J)** Sequencing of the *NR2E3* gene shows no deleterious variants in the ND Control line.

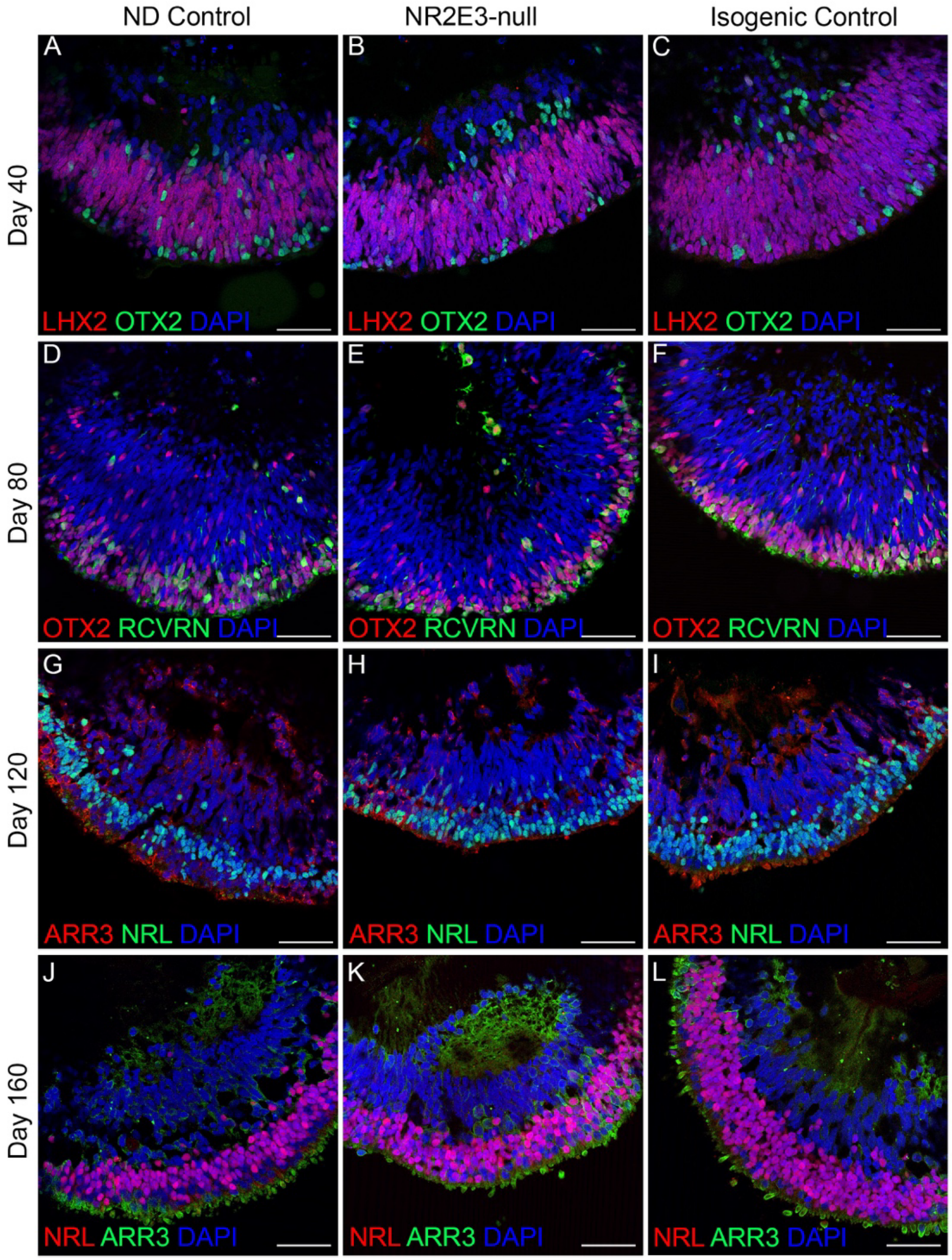

Figure S2. Retinal organoid expression of normal differentiation marker genes. A-C) Organoids from all three lines express *LHX2* and *OTX2*, markers of early neural commitment. **D-F)** By D80 of differentiation, the pan-photoreceptor marker *RCVRN* is expressed in organoid cells. **G-I)** At D120 of differentiation, the cone-specific arrestin *ARR3* and rod-specific factor *NRL* are expressed in the outer photoreceptor layer of organoids. **J-L)** By D160, organization of cone and rod photoreceptor layers is observed. Scalebars represent 50 µm.

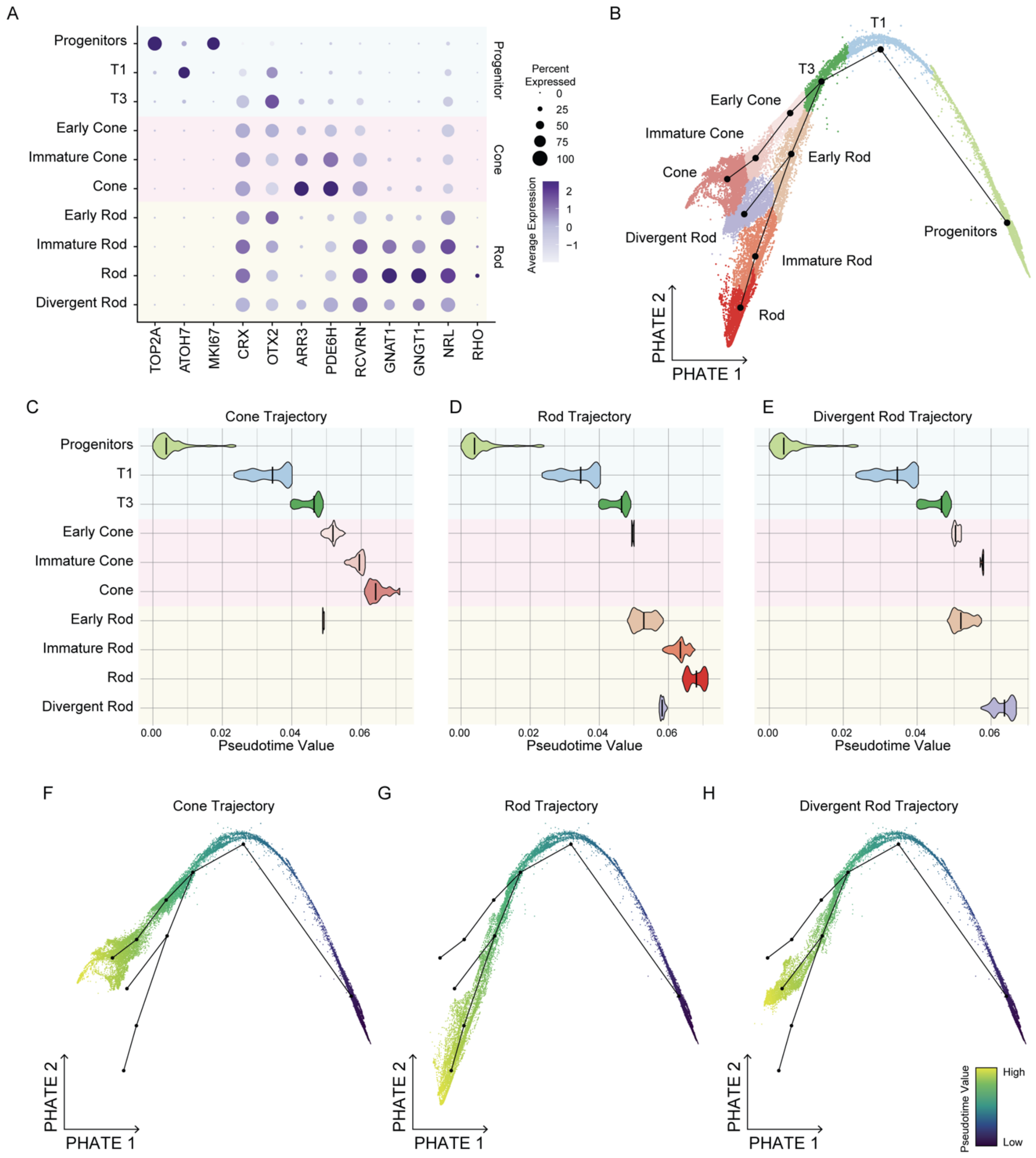

Figure S3. PHATE reduction and trajectory analysis of photoreceptor lineage cells. A) Clusters identified following PHATE reduction express appropriate progenitor-, cone-, and rod-specific genes. **B)** Annotated cells are shown based on PHATE dimensionality reduction. Branching lineages connecting clusters as identified by Slingshot are shown. Two branchpoints are identified giving rise to cones and rods and divergent rods respectively. **C-E**) Pseudotime values were generated for each cell using the three lineages identified in **B**. A violin plot is shown for each trajectory, showing the median pseudotime values (bar) for cells annotated for each cluster. Pseudotime values follow annotated clusters in terms of cell type maturity. Progenitor, T1, T3, and Early Rod clusters contain cells with comparable pseudotime values between the Rod and Divergent Rod trajectories (**D, E**). **F-H**) Trajectories based on the lineages in **B** are shown. Cells are colored based on pseudotime value along each trajectory with purple indicating a lower pseudotime value and yellow indicating a higher pseudotime value (arbitrary units).

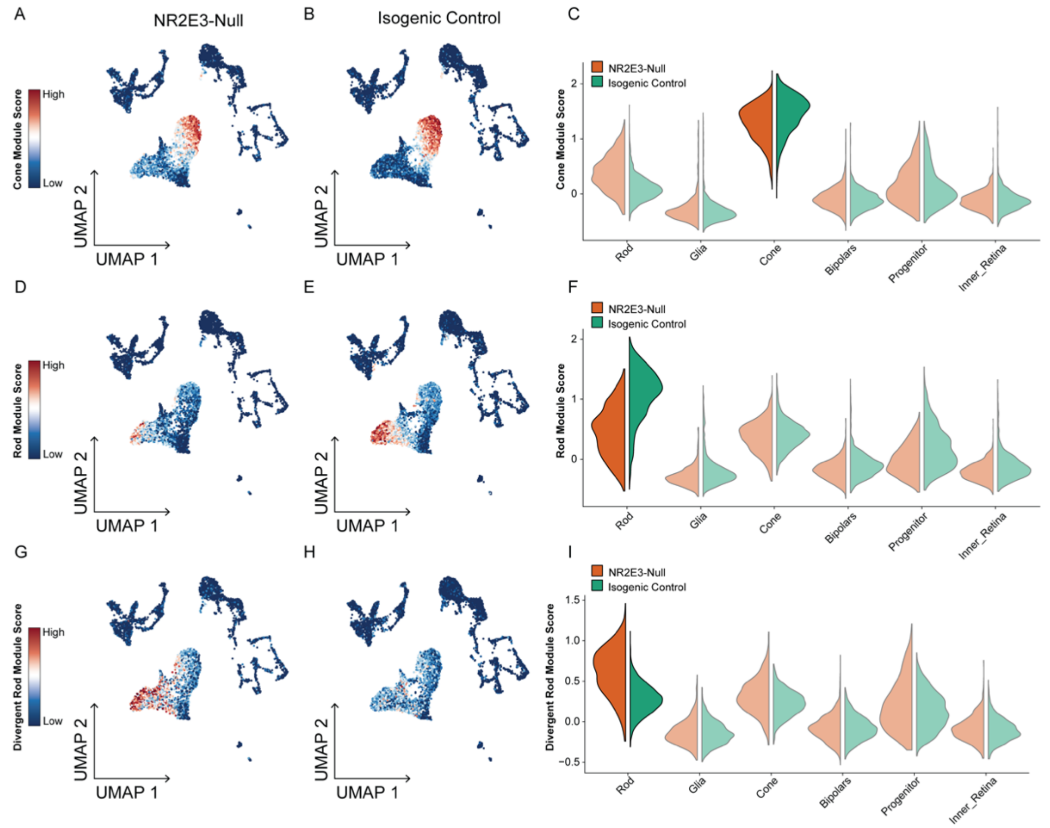

J

Divergent Rod Module	Rod Module	Cone Module
VTN	GNAT1	PDE6H
NR2E3	GNGT1	ARR3
NSMCE2	PDE6G	GUCA1B
SYNDIG1	SAG	GUCA1C
AC112206.2	CABP5	AC112206.2
AC063980.1	RP1	MYL4
ISOC1	NRL	SLC24A2
NEUROG1	ROM1	CC2D2A
AC106798.1	CNGB1	CNGB3
AC055733.2	GNB1	ASIC2
SERPINF1	PPEF2	AC023905.1
GNGT1	GPR160	KCNB2
ROM1	AIPL1	RHOBTB1
RCVRN	PDE6A	DPYSL3
LINC00592	DMD	MPP4
AL391650.1	YBX3	PEX5L
HLA-C	CLUL1	CCSER1
FAM155A	MARCH1	HCN1
FAM174B	PPP2R2B	CAMK1D
TDRD7	AP000462.1	LINC02343
AC104938.1	PCDH15	LRRC39
KCNG4	FAM161A	PRR16
GUCA1B	ANK3	NXNL1
PAG1	USH2A	RTBDN
WWC1	PRPH2	PPP4R4

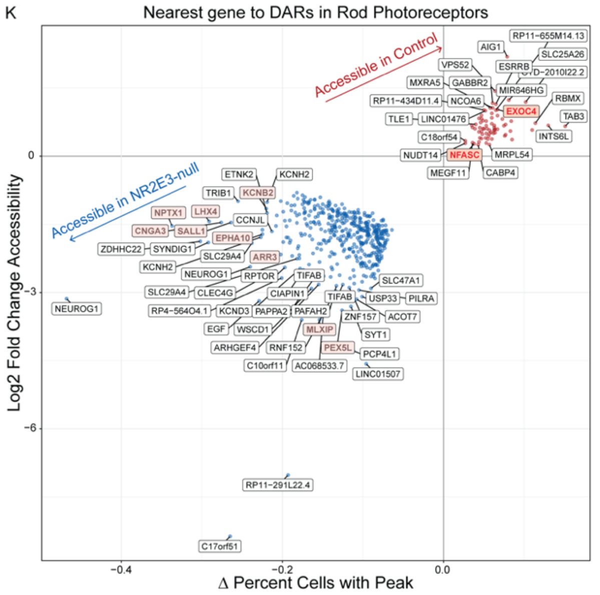

Figure S4. Identification of divergent rods in multimodal sequencing data. A-B) Cells assayed by multimodal sequencing are colored based on enrichment score for the cone gene module, with red indicating higher score. **C)** Cone gene module scores are plotted by cell type split by iPSC line of origin. Both NR2E3-null and Isogenic Control lines generate cones with high cone module score. **D-F)** As in **A-C**, cells are shown with a rod gene module score. There is notable enrichment of the rod module score in Isogenic Control rods versus NR2E3-null rods. **G-I**) The rod cluster of the NR2E3-null line shows the highest gene module score for the Divergent Rod module. **J)** Genes used to construct gene modules used in A-I. **K)** Differentially accessible regions between NR2E3-null and isogenic control rod photoreceptors (i.e., those from Figure 3D) are shown labeled with the symbol of the nearest gene. Within regions preferentially accessible in the NR2E3-null cells (blue dots), cone-specific genes are highlighted in pink. In regions preferentially accessible in control cells (red dots), rod-specific genes are highlighted in red.

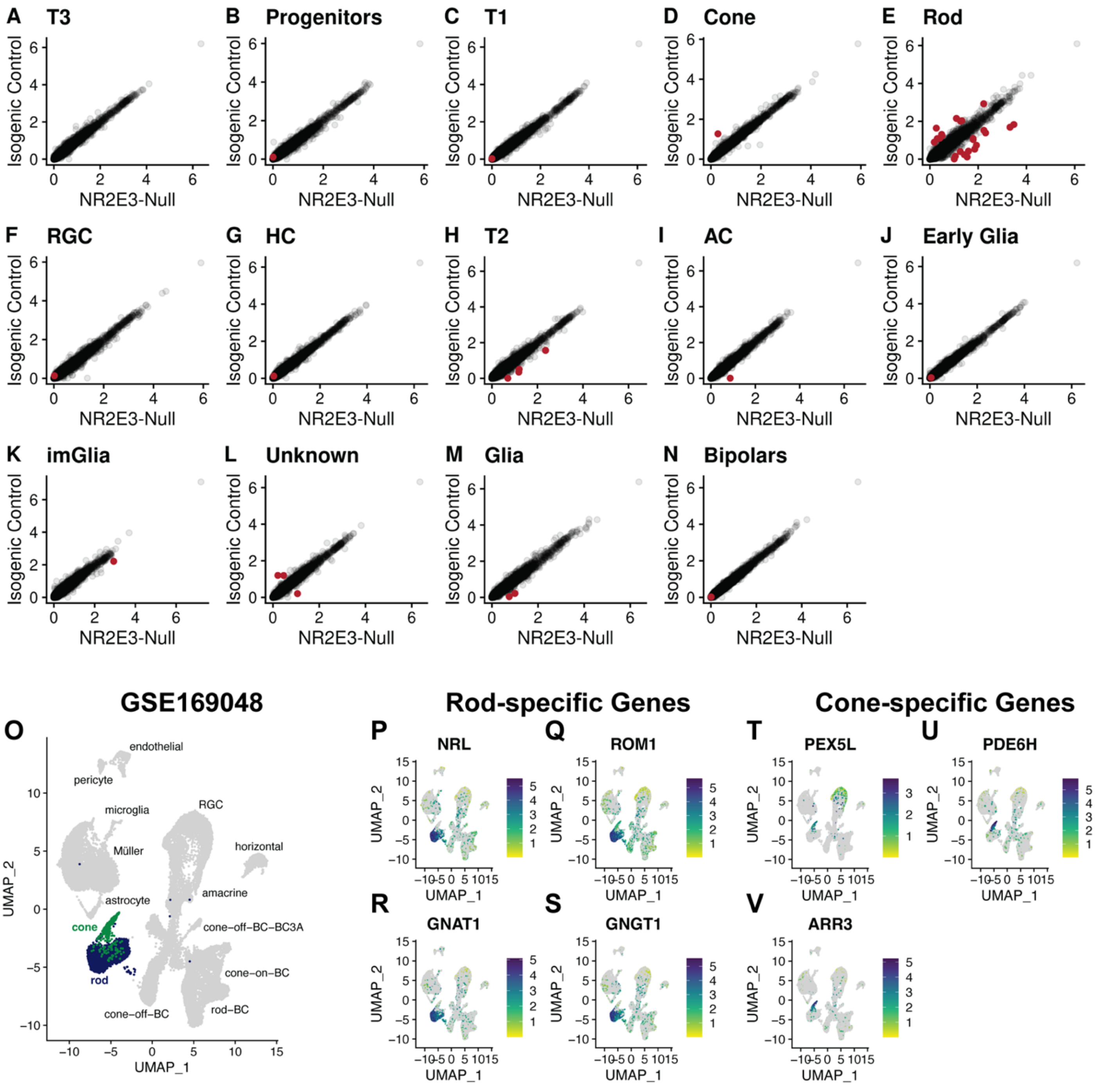

Figure S5. Gene expression in retinal organoid and human donor retinal cells. A-N) Dysregulation of gene expression in the NR2E3-null is restricted to rod photoreceptors. For each cell type, average expression level of every gene is shown on a log1p scale between the NR2E3-null and Isogenic Control lines. Significantly differentially expressed genes (i.e. Log2(FC) > 1 and delta % cells expressing > 10%) are shown in red. **O)** UMAP of single cells from human donor neural retinal cell types assayed by scRNASeq. Data was accessed from GSE169048. Rod and cone photoreceptors are indicated in blue and green, respectively. **P-S)** Expression of *NRL, ROM1, GNAT1,* and *GNGT1* is restricted to human rod photoreceptors. **T-V)** Expression of *PEX5L, PDE6H,* and *ARR3* is restricted to human cone photoreceptors.

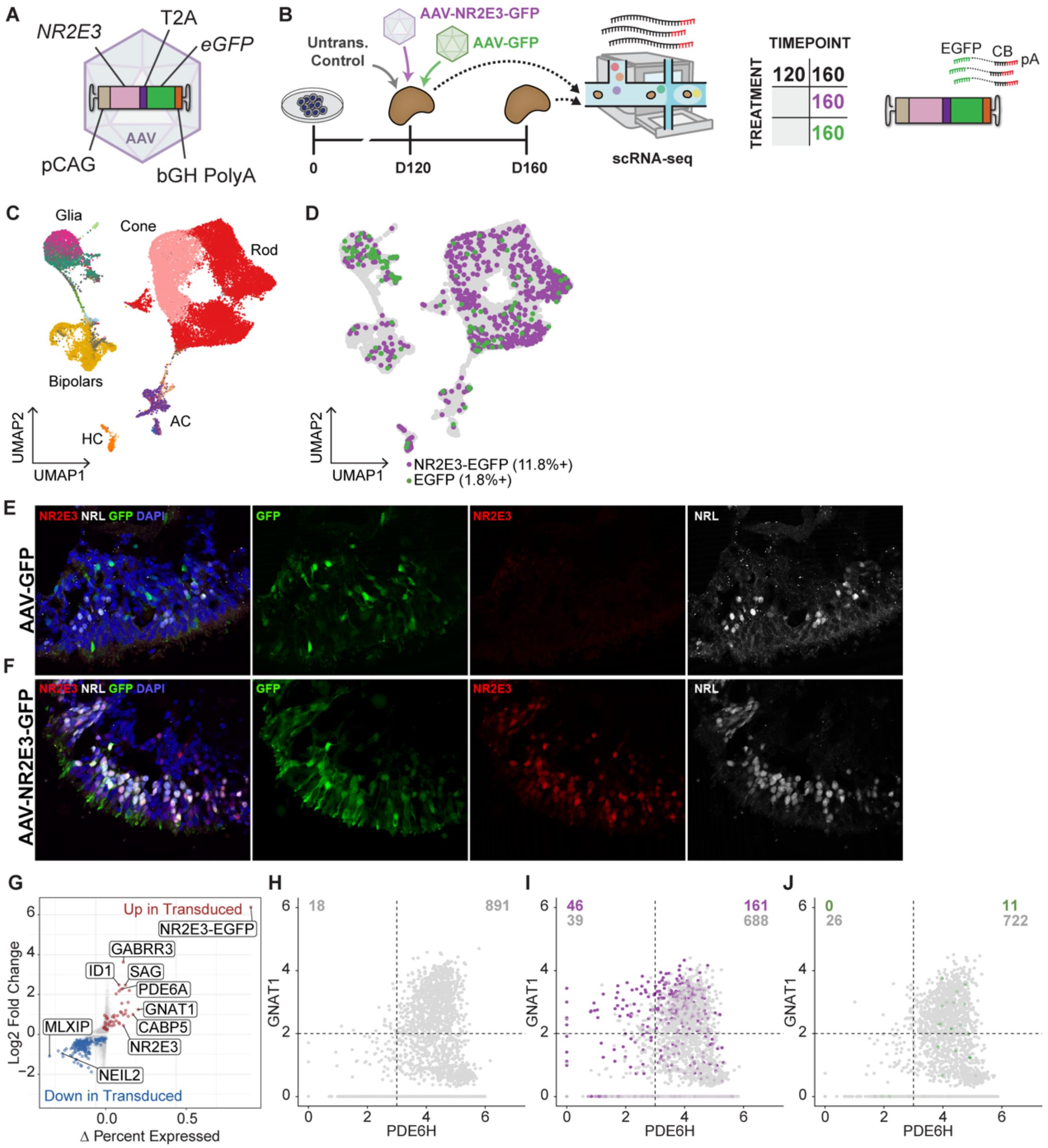

Figure S6. Most divergent rods are refractory to developmental rescue by NR2E3 supplementation. A) AAV-NR2E3-T2A-EGFP was constructed to constitutively express wildtype NR2E3 cDNA and eGFP under the CAG promoter. **B)** NR2E3-null organoids were transduced at D120 with AAV carrying the NR2E3-eGFP construct or AAV carrying eGFP alone. At D160, transduced and untransduced organoids were collected. scRNAseq libraries were prepared and sequenced. Reads mapping to EGFP allow identification of transduced cells in either condition. **C)** UMAP project of gene expression of all four samples from **B)**. Cell types are annotated. **D)** Cells transduced with AAV-NR2E3-EGFP or AAV-GFP are shaded in purple or green, respectively. **E, F)** Transduced cells in AAV-GFP or AAV-NR2E3-EGFP are shown in green. AAV-NR2E3-EGFP transduction restores nuclear NR2E3 staining not observed in AAV-GFP treatment. NR2E3 expression restoration is observed in NRL+ nuclei. **G)** Results of differential gene expression analysis between transduced and untransduced rod photoreceptors from the AAV-NR2E3-EGFP treated sample. The top differentially expressed genes are labeled. **H)** Divergent rods largely co-express GNAT1 and PDE6H. Few cells express GNAT1 without PDE6H expression (dashed box). **I)** A small number (46) of GNAT1-expressing cells display low levels of PDE6H expression in the AAV-NR2E3-EGFP sample. These cells are largely positive for GFP (shown shaded in purple), indicating transduction with AAV-NR2E3-EGFP. Many GFP+ cells (161) also express PDE6H. **J)** Few PDE6H-, GFP+ cells are observed in the sample transduced with AAV-GFP.

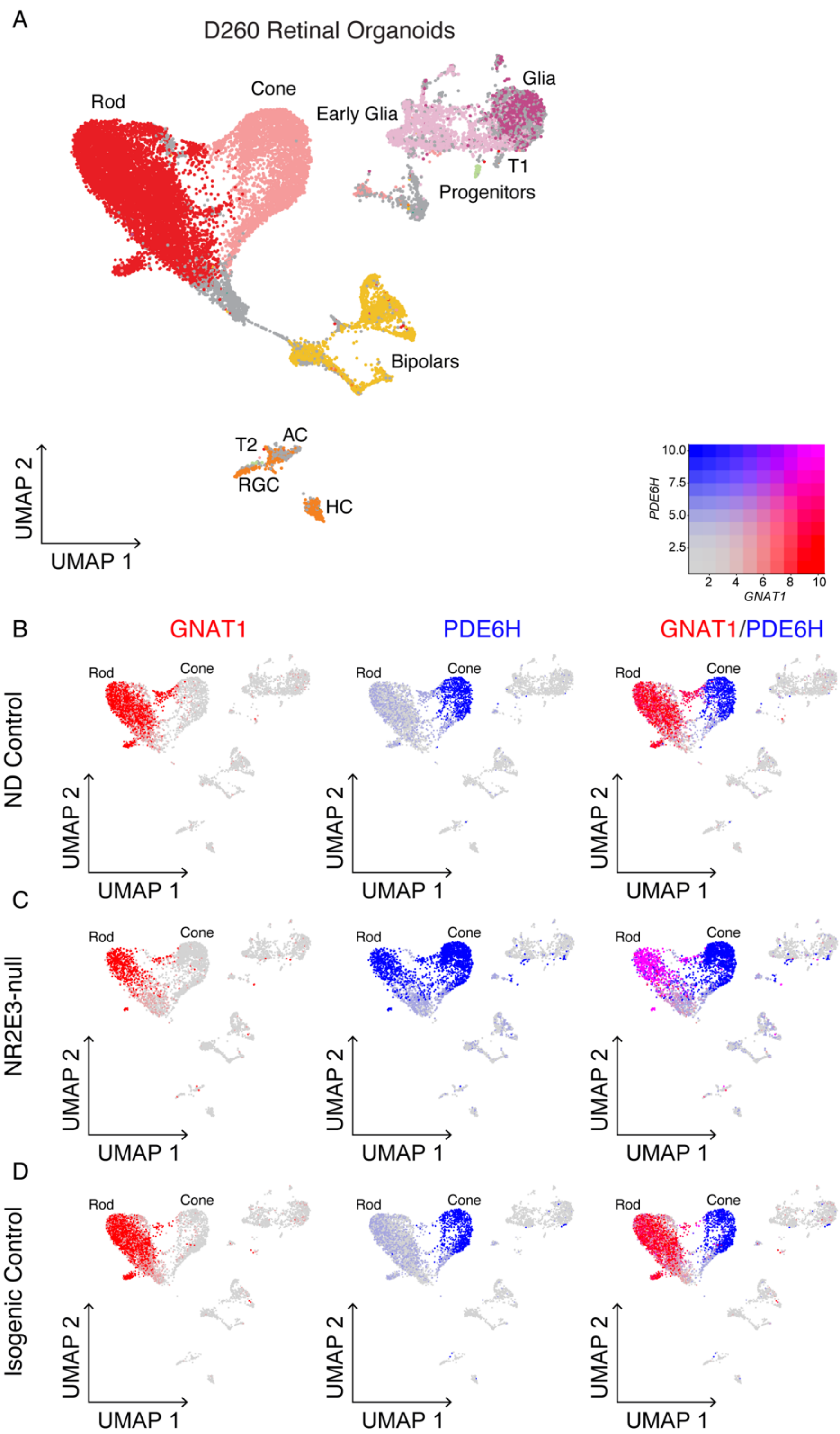

Figure S7. scRNAseq in D260 retinal organoids shows persistence of divergent rods. A) Cells from ND-control, NR2E3-null, and Isogenic Control lines collected at D260 of differentiation. Cells are projected in 2D space based on UMAP reduction derived from gene expression data. Late-stage organoids contain primarily photoreceptor, Müller glia, and bipolar cells. **B)** GNAT1, and PDE6H expression co-segregated into rod and cone photoreceptors in the ND control line. **C)** NR2E3-null organoids contain a population of photoreceptors that co-express GNAT1 and PDE6H (shown in pink, right panel). These represent the Divergent Rods described in the D40-D160 dataset. **D)** Monoallelic correction of NR2E3 restores normal segregation of GNAT1 and PDE6H expression in rod and cone photoreceptors.

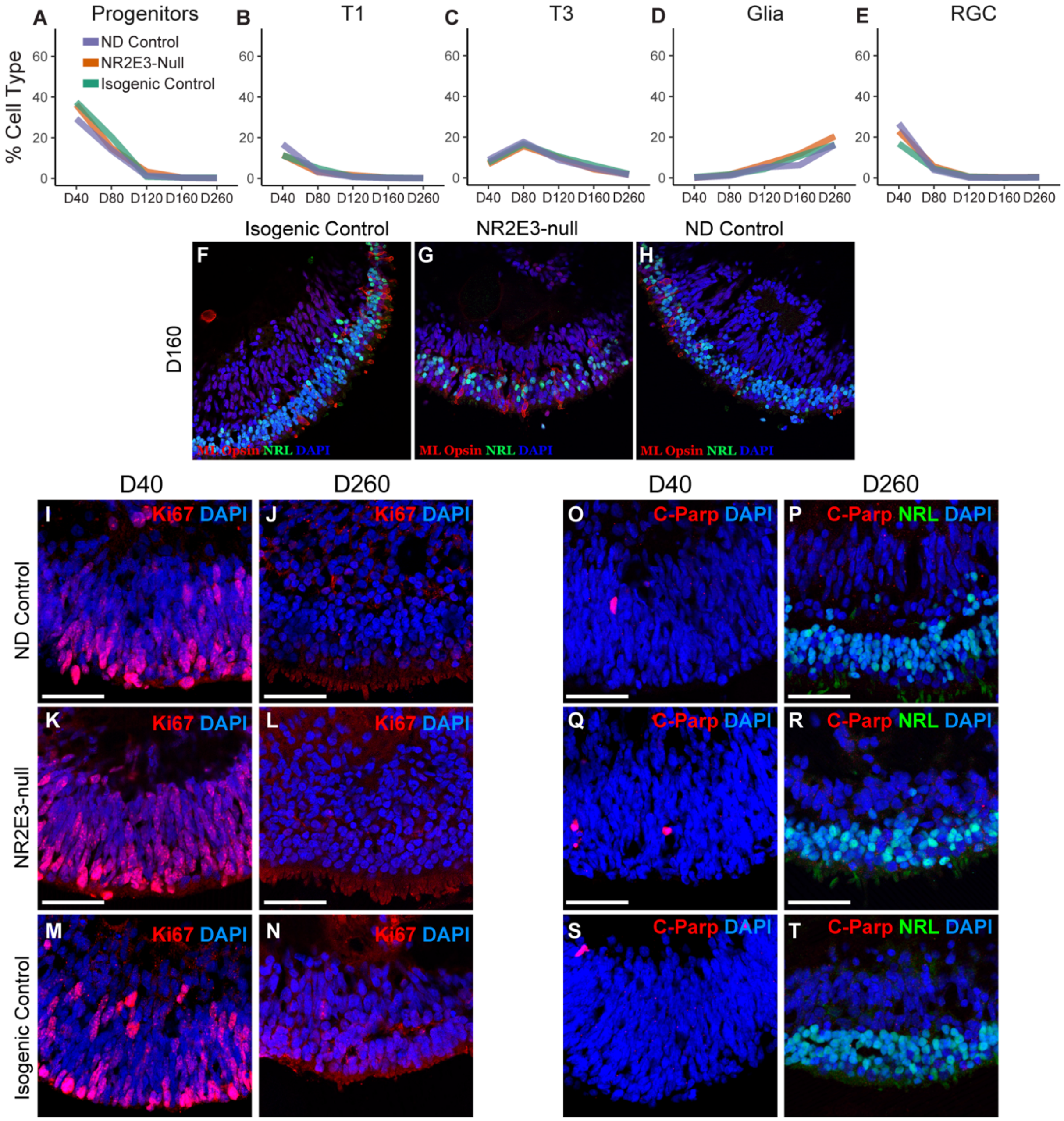

Figure S8. Organoid composition across D40-D260 of differentiation. A-E) The proportion of each cell type per timepoint within sampled organoids is shown. Between D160 and D260, no major differences are observed between lines. **F-G)** D160 organoids stained for the rod transcription factor NRL and the cone M/L-opsin. NR2E3-null (**H**) organoids do not contain a markedly greater number of M/L-opsin-positive cells than control organoids. **I-N)** The marker of proliferation Ki67 is expressed in D40 organoids and lost at D260 in all three lines, indicate no prolongation of proliferation in NR2E3-null organoids. **O-T)** No increase in cleaved PARP is observed in NR2E3-null organoids, indicating no increased apoptosis.

Table S1. Antibodies used.

**Primaries**			
ARR3	Rabbit	LifeSpan Bio	LS-C368677
CHX10	Sheep	Exalpha	X1179P
Cleaved-PARP	Rabbit	Cell Signaling	5625
GNAT1	Rabbit	Thermo	PA5-28336
Ki67	Rabbit	Abcam	AB15580
LHX2	Rabbit	Abcam	AB184337
ML opsin	Rabbit	Millipore	AB5405
Nanog	Goat	R&D Systems	AF1997
NR2E3	Mouse	R&D	PP-H7223-00
NRL	Goat	R&D Systems	AF2945
OCT4	Rabbit	Stemgent	09-0023
OTX2	Goat	R&D Systems	AF1979
PDE6H	Mouse	Santa Cruz	SC-166350
Recoverin	Rabbit	Millipore	AB5585
Rhodopsin	Mouse	Millipore	MAB5316
S opsin (organoid)	Rabbit	Millipore	AB5407
S opsin (donor)	Goat	Santa Cruz	SC-14363
SNCG	Mouse	Abnova	H00006623-MO1
**Secondaries**			
Donkey anti-mouse 488		Thermo	A21202
Donkey anti-goat 488		Thermo	A11055
Donkey anti-sheep 647		Thermo	A21448
Donkey anti-rabbit 647		Thermo	A31573
